# Construction and experimental validation of an acetylation-related gene signature to evaluate the recurrence and immunotherapeutic response in early-stage lung adenocarcinoma

**DOI:** 10.1186/s12920-022-01413-7

**Published:** 2022-12-11

**Authors:** Haiqiang Wang, Xiyan Lu, Jiakuan Chen

**Affiliations:** 1 Department of Thoracic Surgery, The Second Affiliated Hospital of Air Force Military Medical University, No. 1 Xinsi Road, Baqiao District, Xi’an, 710038 Shaanxi China; 2 Department of Traditional Chinese Medicine, The Second Affiliated Hospital of Air Force Military Medical University, No. 1 Xinsi Road, Baqiao District, Xi’an, 710038 Shaanxi China

**Keywords:** Acetylation, Early-stage lung adenocarcinoma, Recurrence, Prognosis, Immunotherapy

## Abstract

**Background:**

Acetylation is a reversible epigenetic process, playing an important role in the initiation and progression of malignant tumors. However, the prognosis value of acetylation-related genes in the early-stage lung adenocarcinoma (LUAD) remains obscure.

**Materials and methods:**

The acetylation-related genes were collected and clustered based on transcriptome sequencing of the patients with early-stage LUAD from the Cancer Genome Atlas. The genomic divergence analysis, protein–protein interaction network construction, Lasso regression, and univariate Cox regression were used to identify the significant biomarkers for the recurrence of the early-stage LUAD. The multivariate Cox regression was used to establish the predictive model. Gene Expression Omnibus was systemically retrieved and four independent datasets were used for external validation. 23 early-stage LUAD samples were collected from the local hospital to detect the expression difference of the genes in the model. Transfection assays were performed to verify the regulatory ability of the screened gene to the proliferation of LUAD cell lines. The single-cell RNA sequencing of the early-stage LUAD patients and two lung cancer cohorts receiving immunotherapy were utilized to explore the predictive ability of the established model to immunotherapeutic sensitivity.

**Results:**

The clustering based on acetylation-related genes was significantly associated with the recurrence (P < 0.01) and immune infiltration statuses. Through a series of bioinformatical and machine learning methods, RBBP7 and YEATS2 were ultimately identified. Accordingly, a novel gene signature containing RBBP7 and YEATS2 was developed to evaluate the recurrence-free survival of early-stage LUAD, which was then validated in five independent cohorts (pooled hazard ratio = 1.88, 95% confidence interval = 1.49–2.37) and 23 local clinical samples (P < 0.01). The knock-down of YEATS2 obviously suppressed proliferation of H1975 and HCC-827 cells. Single-cell RNA sequencing analyses indicated that RBBP7 and YEATS2 were both associated with the tumor immune response, and the prognosis signature could predict the immunotherapeutic response in two cohorts receiving immunotherapy (P < 0.05; P < 0.01).

**Conclusions:**

Totally, an acetylation-related gene signature is constructed, helping to evaluate the recurrence and immunotherapeutic effectiveness of early-stage LUAD patients.

**Supplementary Information:**

The online version contains supplementary material available at 10.1186/s12920-022-01413-7.

## Introduction

Lung cancer is the second commonest cancer and the leading cause of cancer-specific death globally [1]. Lung adenocarcinoma (LUAD), characterized by early recurrence and high mortality, is the primary pathological subtype of lung cancer [2]. Despite the fact that tremendous progress has been made in the diagnostic and therapeutic methods, the 5-year overall survival of LUAD is still poor [3]. Many clinical traits have demonstrated the effectiveness of immune checkpoint inhibitors (ICIs) in LUAD, but the durable response can only be observed in a minority of subjects [4]. Additionally, accreting to statistics, almost 30–45% of early-stage LUAD relapse within five years after tumor resection [5]. The high recurrence rate and immunotherapeutic response uncertainty of early-stage LUAD bring unfavorable prognosis, much suffering, and a high economic burden to the patients. Hence, it is urgently demanded to develop a clinical tool to evaluate the immunotherapeutic response and recurrence of early-stage LUAD, which can guide individualized treatment.

Acetylation, a chemical process modifying the acetyl of proteins and mRNAs [6], is widely involved in various cellular life activities, such as transcription, chemotaxis, cell signal transduction, stress response, protein hydrolysis, and apoptosis [7]. Acetylation plays a vital role in tumorigenesis, especially in metabolism [8], DNA damage response [9], autophagy [10], proliferation [11], and differentiation [12]. Acetylation is also essential in tumor immunity. For example, PCAF, a member of histone acetyltransferase, can interact with Tregs [13], M1 macrophages [14], and T cells [13], to perform antitumor functions. Many studies have reported the critical role of acetylation in early tumorigenesis. For instance, Park and his colleagues found that the acetylation of p53 was associated with the malignant phenotypes in early-stage hepatocellular carcinoma [15]. The expression of SIRT2, an acetylation regulator, was obviously elevated in many early-stage tumors originating in various organs [16]. The loss of H4K16ac, an important histone acetylation mark, was an early sign to breast cancer [17]. Acetylation was also reported to be associated with tumor relapse. The hypoacetylation of histone H3 at K9, K18 and H4K16 was strongly associated with the recurrence of prostate cancer [18]. Previous studies have reported some vital acetylation-related genes in LUAD [19–21], but the comprehensive study on the roles of acetylation-related genes in LUAD, especially in early-stage LUAD, is devoid.

Herein, we retrieved the acetylation-related genes from the Molecular Signatures Database (MSigDB). The transcriptome sequencing data of the patients with early-stage LUAD was comprehensively collected from the Cancer Genome Atlas (TCGA) and Gene Expression Omnibus (GEO) as the training and external validation datasets, respectively. The unsupervised clustering was performed to detect the association of acetylation-related genes with recurrence and immune infiltration levels. A series of bioinformatical analyses methods were adopted to develop a predictive gene signature for the recurrence-free survival (RFS) rate of the patients with early-stage LUAD, and its clinical usefulness was then confirmed in a meta-analysis and clinical samples from the local hospital. The single-cell RNA sequencing (scRNA-seq) of the patients with early-stage LUAD and the RNA-sequencing (RNA-seq) of the lung cancer patients receiving immunotherapy were used to investigate the potential of the established gene signature to predict the immunotherapeutic effectiveness. Our study provides a promising tool to evaluate the recurrence of the early-stage LUAD and the immunotherapeutic response, which also offers underlying cut-in points for the molecular mechanism research from the aspect of acetylation.

## Materials and methods

### Data collection and processing

240 acetylation-related genes were retrieved from MSigDB (https://www.gsea-msigdb.org/gsea/msigdb/, accessed on March 19, 2022), as shown in Additional file 1: Table S1. The transcriptome sequencing data of 334 early-stage LUAD patients (tumor-node-metastasis, TNM, stage I-II) and 59 adjacent normal lung samples, the corresponding clinical traits, and their RFS information were obtained from TCGA (https://portal.gdc.cancer.gov/, accessed on March 20, 2022), as the training dataset. We queried the GEO website (https://www.ncbi.nlm.nih.gov/geo/, accessed on March 21, 2022) to download the external validation datasets using the keyword “lung cancer”. The filtering criteria were viewed below: 1) The dataset must include the transcriptome sequencing data of the patients with early-stage LUAD. 2) The recurrence statuses and follow-up duration are available in the GEO database or the supplementary materials of the original article.

Additionally, the datasets associated with immunotherapy were also retrieved in the GEO database with the keywords “lung cancer”, and the filtering criteria include: 1) The patients with lung cancer have received anti-PD-1/PD-L1 or other ICIs treatment. 2) The dataset contains the transcriptome sequencing data of the lung cancer subjects. 3) The follow-up information or response to the ICIs has been recorded and publicly available. Given the limited numbers of immunotherapy-related datasets, we took all lung cancer patients, not just early-stage LUAD patients, into consideration.

After the careful and manual review of all the records in the GEO, four early-stage LUAD-related datasets were identified, including GSE30219, GSE31210, GSE37745, and GSE5008. In addition, two immunotherapy-related datasets containing GSE126044 and GSE13522 were also incorporated into the study. Their gene expression matrices were directly downloaded from the GEO. The detailed information of these datasets is displayed in Table 1.

**Table 1:** The detailed information of the public datasets downloaded from GEO

ID	Experimental type	Platform	Samples (control/tumor)	Region
GSE30219	Microarray	GPL570	14/293	France
GSE31210	Microarray	GPL570	20/226	Japan
GSE37745	Microarray	GPL570	0/196	Sweden
GSE50081	Microarray	GPL570	0/181	Canada
GSE135222	RNA-seq	GPL16791	0/27	South Korea
GSE126044	RNA-seq	GPL16791	0/16	South Korea
GSE198291	Single-cell RNA-seq	GPL16791	8/8	China

*GEO* gene expression omnibus

The RNA-seq data used in this research was first normalized into transcripts per kilobase of exon model per million mapped reads (TPM) format, and the gene sequencing data from microarray experiments were all log2 transformed. The mean expression value would be adopted if a gene corresponded to multiple probe IDs. Furthermore, the early-stage LUAD patients with less than three months of follow-up duration would be excluded.

### Clinical sample collection

The adjacent normal tissues and the LUAD tissues from 23 patients undergoing partial/radical pulmonary resection were collected between April 2021 and March 2022 in the Second Affiliated Hospital of Air Force Military Medical University. The fresh adjacent normal and the tumor samples were isolated from the site > 2 cm away from the tumor edge and the center of the tumor during surgery, respectively, and then stored in liquid nitrogen for RNA extraction. All the subjects enrolled did not undergo preoperative chemotherapy, immunotherapy, or radiotherapy. The TNM stage information of the LUAD samples was determined based on the eighth TNM staging system defined by the American Joint Commission on Cancer. The clinicopathological features of the 23 patients were displayed in Additional file 2: Table S2.

The protocol of this study has been approved by the Ethics Committee of the Second Affiliated Hospital of Air Force Military Medical University (ID: 2021-KY-03191), and this study was performed in accordance with the principles of the Declaration of Helsinki. All the patients have signed the informed consent.

### Real-time quantitative PCR experiments

The total RNA of the clinical samples was isolated with the Trizol-chloroform method (Thermo Fisher Scientific, China), and the concentration of RNA was measured by NanoDrop spectrophotometer (Thermo Fisher Scientific, USA). Following the manufacturer’s protocol, we conducted the real-time quantitative PCR (RT-qPCR) with PrimeScript RT Reagent Kit (Takara, China) and SYBR Premix ExTaq kit (Takara, China). The reaction condition was set as follows: 95 ℃/5 min, 1 cycle; 95 °C/10 s; 60 °C/30 s; 72 °C/30 s; a total of 40 cycles. GAPDH was chosen as the internal reference for RBBP7 and YEATS2. The relative expression values were calculated based on the 2^−ΔΔCt^ method. All the experiments were repeated 3 times and the mean value was adopted. The primer sequence used in this experiment was shown in Table 2.

**Table 2:** The primer sequence used in this study

Gene	Primer sequence (5′–3′)
RBBP7	F: GAGGAGCGTGTCATCAATGAA
	R: GCATGGGTCATAACCAGGTCATA
YEATS2	F: CCATCAAAGAAACCGACCCTG
	R: CTTCAATCAGTCGCTGGTCAAT
GAPDH	F: GGAGCGAGATCCCTCCAAAAT
	R: GGCTGTTGTCATACTTCTCATGG

### Cell lines and RNA interference

Four human LUAD cell lines, including A549, HCC827, H3255 and H1975, and one human immortalized pulmonary alveolar epithelial cell line (HPAEpiC), which were all obtained from the Cell Bank of the Type Culture Collection of the Chinese Academy of Sciences (Shanghai, China), were used. The cells were cultured in RPMI 1640 medium (Gibco, China), which was supplemented with 10% fetal bovine serum (Gibco, China), 100 U/mL penicillin, and 100 mg/mL streptomycin. The YEATS2 small interfering RNA (si-YEATS2) and the control siRNA (si-NC) were designed and synthesized by Biosyntech (Suzhou, China). Lipofectamine 3000 (Invitrogen, USA) was used to conduct the transfection assay according to the manufacturer’s instructions. RT-qPCR was implemented to detect the transfection efficacy.

### Cell counting kit-8 assay

Cell proliferation ability was detected by cell counting kit-8 (CCK-8, GLPBIO, USA). The cells were cultivated at 96-well plates at a density of 1000–10,000 cells/well for 24 h at a humidified atmosphere with 5% CO_2_, and a 100-μl aliquot of cell suspension was seeded to each plate. 2 vice-holes were set in each experiment, and the mean of the optical density (OD) values of 3 holes were adopted. The cell numbers were evaluated every 24 h. In each test, 10 μl CCK-8 reagent was added to each plate, and the plate was incubated for 2 h. Afterwards, the OD values were detected at 450 nm using a microplate reader. All the experiments repeated 3 times to conduct the statistical analyses.

### Acquisition and processing of scRNA-seq data

The transcriptome data from the single-cell level were extracted from GSE198291 in the GEO database, and the associated information is shown in Table 1. 720 cell samples from eight early-stage LUAD patients contained 520 cell samples from tumor tissue (TT), 184 cell samples from paratumor tissue (PT), and 16 cell samples from negative control (NC) samples. PT was defined as the site more than 0.5 cm away but less than 2 cm away from the tumor edge, and NC samples were extracted outside 2 cm around the tumor. Seurat package of R software (version 4.1.0) was utilized to process the single-cell data of the cell samples from TT. We excluded the genes which have been detected in less than three cells. Additionally, the cells with less than 200 genes detected and more than 5% of mitochondrial genes would also be eliminated. Then the data were normalized, and the Top 1500 variable genes were determined. Principal component analysis (PCA) was performed to reduce the dimension of the scRNA-seq matrix preliminarily, and the genes with a high correlation with each principal component (PC) were identified. The Top 20 PC were included in the following analyses. Subsequently, t-distributed stochastic neighbor embedding (t-SNE) was conducted to divide the cells into different clusters, and the markers genes of each cell cluster were screened with |logFC|> 0.5 and adjusted P-value < 0.05 filtering. CellMarker (http://bio-bigdata.hrbmu.edu.cn/CellMarker/, accessed on March 25, 2022), which was a database of different cell markers of human tissue, and CancerSEA (http://biocc.hrbmu.edu.cn/CancerSEA/, accessed on March 25, 2022), which focused on the biomarkers of cancer functional states, were both used for cell-type annotation based on the cell markers.

### Unsupervised clustering

The consensus clustering was conducted via the ConsensusClusterPlus package in R software to divide the early-stage LUAD cases into different subgroups. The optimal k value, equaling the subgroup numbers, was determined by cumulative distribution function (CDF) curves.

### Gene Set Enrichment Analysis

Gene Set Enrichment Analysis (GSEA) was conducted via the GESA software (version 4.1.0) downloaded from the GSEA official website (https://www.gsea-msigdb.org/gsea/). The permutation type was set to “gene_set”, and other settings were left on the default parameters. Hallmark gene sets (version 7.5.1), obtained from the MSigDB database, were chosen as the reference. The gene signature with |normalized enrichment score (NES)|> 1, normalized P-value (NOM P) < 0.05, and false discovery rate (FDR) Q-value < 0.05 was considered to be statistically significant.

### Genomic difference analysis and protein–protein interaction network construction

The differentially-expressed genes (DEGs) between the adjacent normal and LUAD samples were identified by the limma package. |logFC|> 1 and FDR < 0.05 were set as the filtering threshold. Afterwards, all the DEGs were uploaded to the STRING database (https://cn.string-db.org/, accessed on March 22, 2022) to construct a protein–protein interaction (PPI) network, and the minimum required interaction score was equal to 0.4. Cytoscape software (version 3.8.0) was used to visualize the PPI network; meanwhile, the importance of the genes in the network was measured by the cytoHubba plug-in in the Cytoscape software.

### Development of the predictive signature

Two different dimension-reduction methods, including least absolute shrinkage and selection operator (Lasso) and univariate Cox regression, were taken to identify the hub genes as the biomarkers for the RFS of early-stage LUAD. Lasso regression was conducted with tenfold cross-validation using the glmnet package in R. The survival R package was utilized to perform the univariate Cox regression, where P-value < 0.05 was considered statistically significant. The genes con-determined by Lasso and univariate Cox regression would be included in the prognosis model via the multivariate Cox analysis, which was also conducted by the survival R package. Here, we christened the risk score calculated by the established model acetylation-related score (ARS), and the ARS of each case was evaluated as indicated below: $ARS={\sum }_{i=1}^{n}{Coefficient}_{i}{\times expression\left(mRNA\right)}_{i}$.

### Survival analysis and meta-analysis

The Kaplan–Meier survival analysis with log-rank test was performed by the survival R package, which was also utilized to calculate the hazard ratio (HR) and its corresponding 95% confidence interval (CI). X-tile software was used to detect the optimal cut-off value, and P-value < 0.05 was significant. Meta-analysis was implemented to clarify the prognosis value of the established model through the Reviewer Manager (version 5.3). The random effects model would be adopted if the heterogeneity test displayed a significant difference (P < 0.05); otherwise, the fixed effects model would be utilized.

### Immunohistochemistry

The immunohistochemical staining of the screened genes in the normal and LUAD samples was obtained from the Human Protein Atlas (https://www.proteinatlas.org/) to detect the distribution and protein expression level of these genes.

### Pan-cancer analysis from the single-cell level

The association of the hub genes with various functional states of malignant cells, including angiogenesis, apoptosis, cell cycle, differentiation, DNA damage, DNA repair, epithelial-mesenchymal transition, hypoxia, inflammation, invasion, metastasis, proliferation, quiescence, and stemness, were detected on the CancerSEA website. The heat map, drawn by the ggplot2 package, was used to visualize the correlation strength.

### Clinical association analysis

The common clinicopathological features, such as gender, age, and TNM stages, were collected from the TCGA as well. All the parameters were transformed into binary variables, and the optimal cut-off value of continuous variables was determined by the X-tile. To explore whether ARS was an independent prognosis predictor, univariate and multivariate Cox regressions were conducted using the survival package after excluding the subjects with missing clinicopathological information.

### Construction and validation of a prognostic nomogram

The nomogram, including ARS and the clinicopathological traits, was constructed via the rms and survival packages. The calibration plot and the receiver operating curve (ROC), which partly reflect the predictive ability of the nomogram, were drawn by the rms, survival, and pROC packages.

### Immune infiltration and immunotherapeutic effectiveness evaluation

Multiple current algorithms, including XCELL, TIMER, QUANTISEQ, MCPCOUNTER, EPIC, CIBERSORT-ABS, and CIBERSORT, were used to evaluate the infiltration level of various immune cells in early-stage LUAD samples from the TCGA. We calculated the Spearman correlation coefficients between the ARS and the immune cell infiltration proportion, and P-value < 0.05 was statistically significant. Additionally, to estimate the immunotherapeutic effectiveness of the early-stage LUAD patients, we downloaded the immunophenoscore (IPS) of each subject with LUAD from the Cancer Immunome Atlas (TCIA, https://tcia.at/home). IPS was developed to predict the therapeutic response of the cancer patients receiving anti-PD-1 or anti-CTLA-4 treatment, and the subjects with higher IPS are more likely to benefit from the ICIs [22].

### Statistical analyses

The statistical analyses of the whole study were based on R software. Unless otherwise specified, Wilcoxon signed-rank test was used to compare the levels of the continuous variables in two groups and the Kruskal–Wallis test was used for comparison when the number of groups > 2. For the categorical variables, Pearson’s chi-square test and Fisher’s exact test were conducted to assess the difference after excluding the missing values. The Spearman-correlation evaluation was performed using the “cor.test” function in R. For the data from the RT-qPCR experiments, paired t-test was used. Students’ t-test was used to compare the difference in the in vitro cell experiments. P-value < 0.05 was considered significant. *P < 0.05, **P < 0.01, ***P < 0.001.

## Results

### The clustering based on acetylation-related genes was associated with recurrence and immune response.

**Fig. 1:**
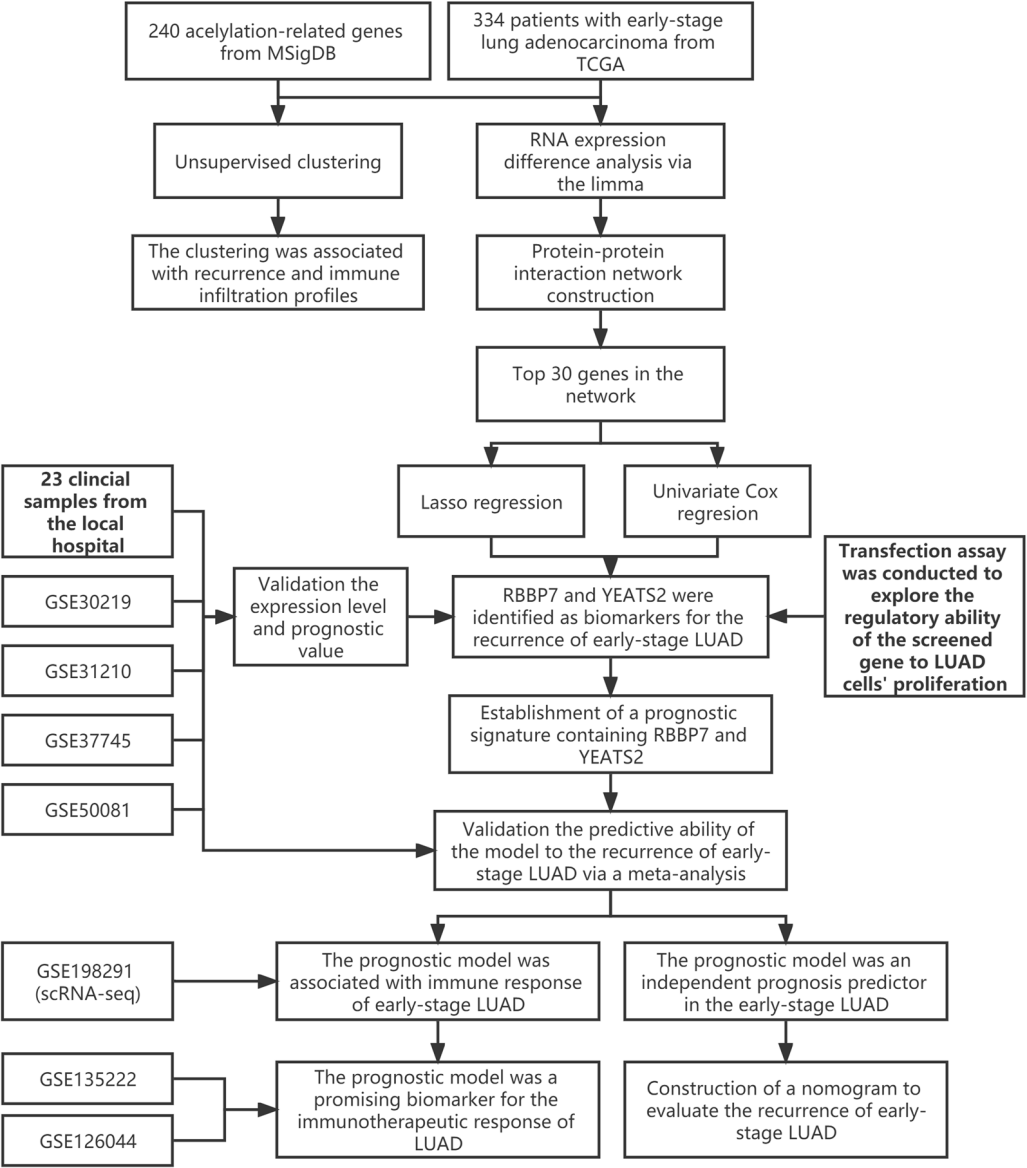
The work flow of the present study

**Fig. 2:**
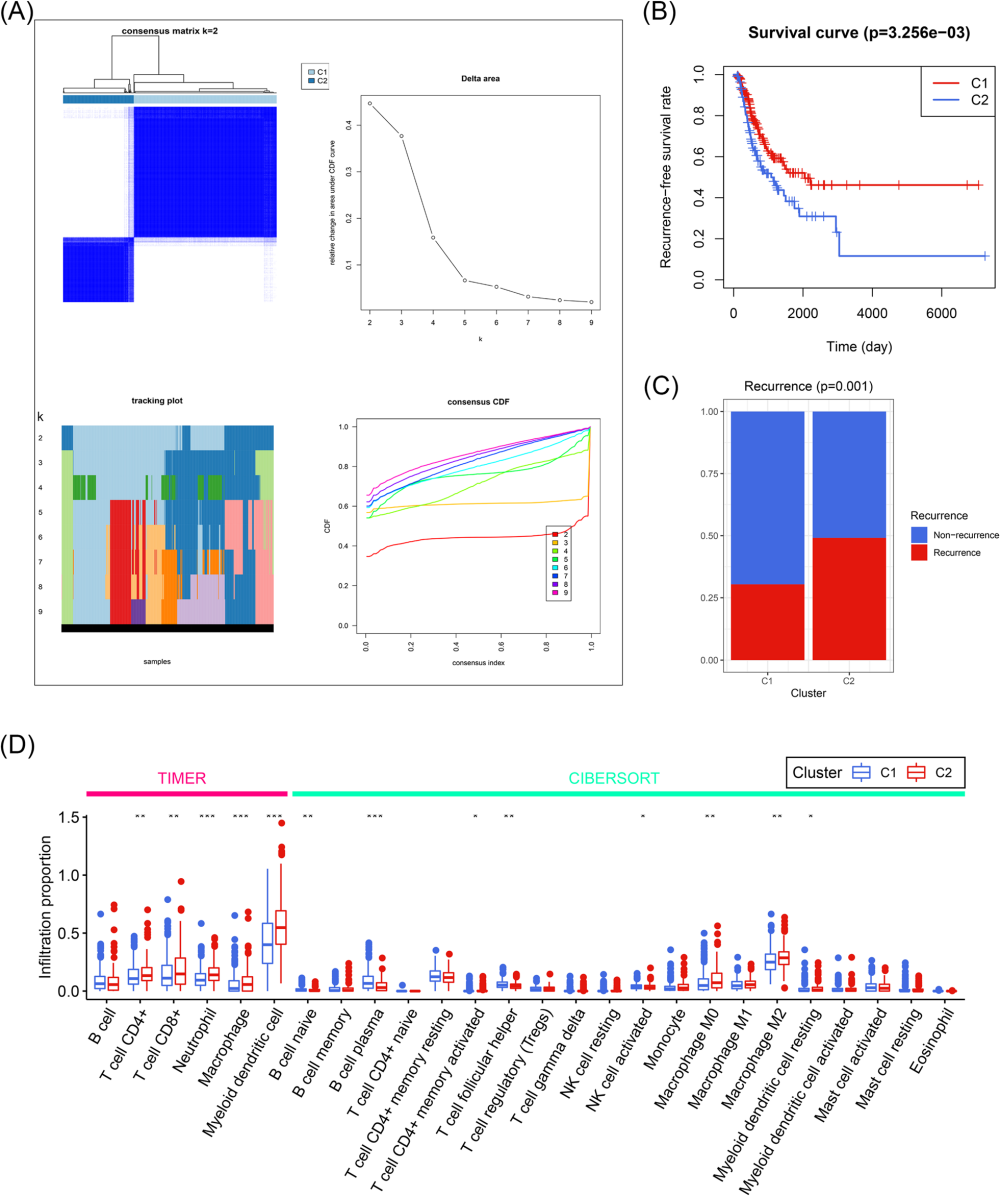
The unsupervised based on the acetylation-related genes were associated with the recurrence and immune infiltration profiles of early-stage LUAD. **A** The early-stage LUAD patients were divided into two groups according to the consensus clustering algorithm. **B** The clustering was associated with the recurrence-free survival rate of early-stage LUAD patients. **C** The tumor of early-stage LUAD patients in C2 was more likely to relapse. **D** The immune cell infiltration levels calculated by TIMER and CIBERSORT algorithms in C1 and C2 patients. *LUAD* lung adenocarcinoma, *C1* Cluster 1, *C2* Cluster 2; *P < 0.05, **P < 0.01, ***P < 0.001

The flow chart of the present study is displayed in Fig. 1. A sum of 334 patients with early-stage LUAD from the TCGA were included in the training dataset. Based on the 240 acetylation-related genes collected from MSigDB, 334 patients were divided into Cluster 1 (C1) or Cluster 2 (C2) through the consensus clustering, as shown in Additional file 3: Table S3 and Fig. 2A. Compared with the patients in the C1, those in the C2 subgroup exhibited worse RFS (P < 0.01, Fig. 2B) and their cancer were more likely to relapse (P < 0.01, Fig. 2C). Additionally, GSEA analysis indicated that the LUAD samples labelled with the C2 group had a stronger immune response (Additional file 4: Fig. S1) and a higher infiltration proportion of multiple immune cells (Fig. 2D, Additional file 5 Table S4), implying the tight correlation between the acetylation process and early-stage LUAD’s recurrence and tumor immune infiltration profiles.

### RBBP7 and YEATS2 were identified as significant biomarkers for the recurrence of early-stage LUAD

**Fig. 3:**
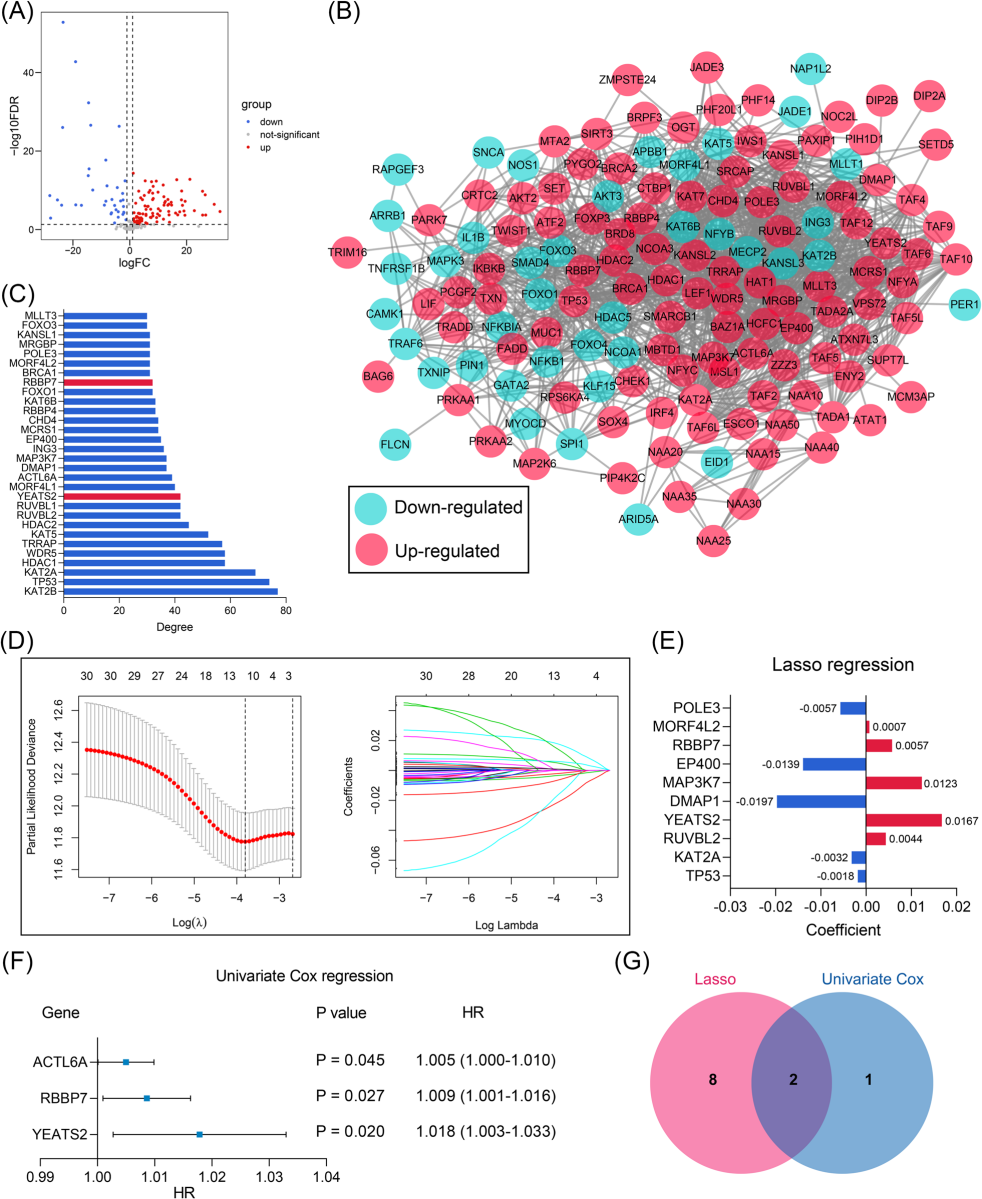
RBBP7 and YEATS2 were identified as the hub genes as the predictive biomarkers for the recurrence of early-stage LUAD patients. **A** The volcano plot indicated that a total of 152 acetylation-related genes were differentially expressed between 59 adjacent normal and 334 early-stage LUAD samples. **B** The PPI network of the DEGs. **C** The Top 30 genes with the highest degree value in the network. **D** Lasso regression identified 10 genes showing significant predictive value to RFS of early-stage LUAD. **E** The coefficients of the 10 genes in the Lasso regression. **F** Three genes were identified by the univariate Cox regression with P-value < 0.05 filtering. **G** RBBP7 and YEATS2 were con-determined by the Lasso regression and univariate Cox regression. *LUAD* lung adenocarcinoma, *PPI* protein–protein interaction, *DEGs* differentially-expressed genes, *RFS* recurrence-free survival, *Lasso* least absolute shrinkage and selection operator

Multiple bioinformatical and dimension-reduction methods were adopted to identify the hub genes associated with the RFS of early-stage LUAD. First, the genomic difference analysis was conducted, and a total of 152 DEGs associated with acetylation were screened, including 43 down-regulated and 109 up-regulated genes (Fig. 3A). Subsequently, the 152 DEGs were uploaded to the STRING database, and the PPI network was constructed (Fig. 3B). According to the cytoHubba app, the Top 30 genes with the highest degree value were determined (Fig. 3C, Additional file 6: Table S5), which were then included in the dimension-reduction analyses. After Lasso regression with tenfold cross-validation, 10 of the Top 30 genes were considered to be significantly associated with the RFS of the early-stage LUAD patients (Fig. 3D), and their coefficients were displayed in Fig. 3E. However, only RB Binding Protein 7, Chromatin Remodeling Factor (RBBP7) and YEATS Domain Containing 2 (YEATS2) of the 10 genes were con-determined by the univariate Cox regression (Fig. 3F, G). The details of the univariate Cox regression are shown in Additional file 7: Table S6.

**Fig. 4:**
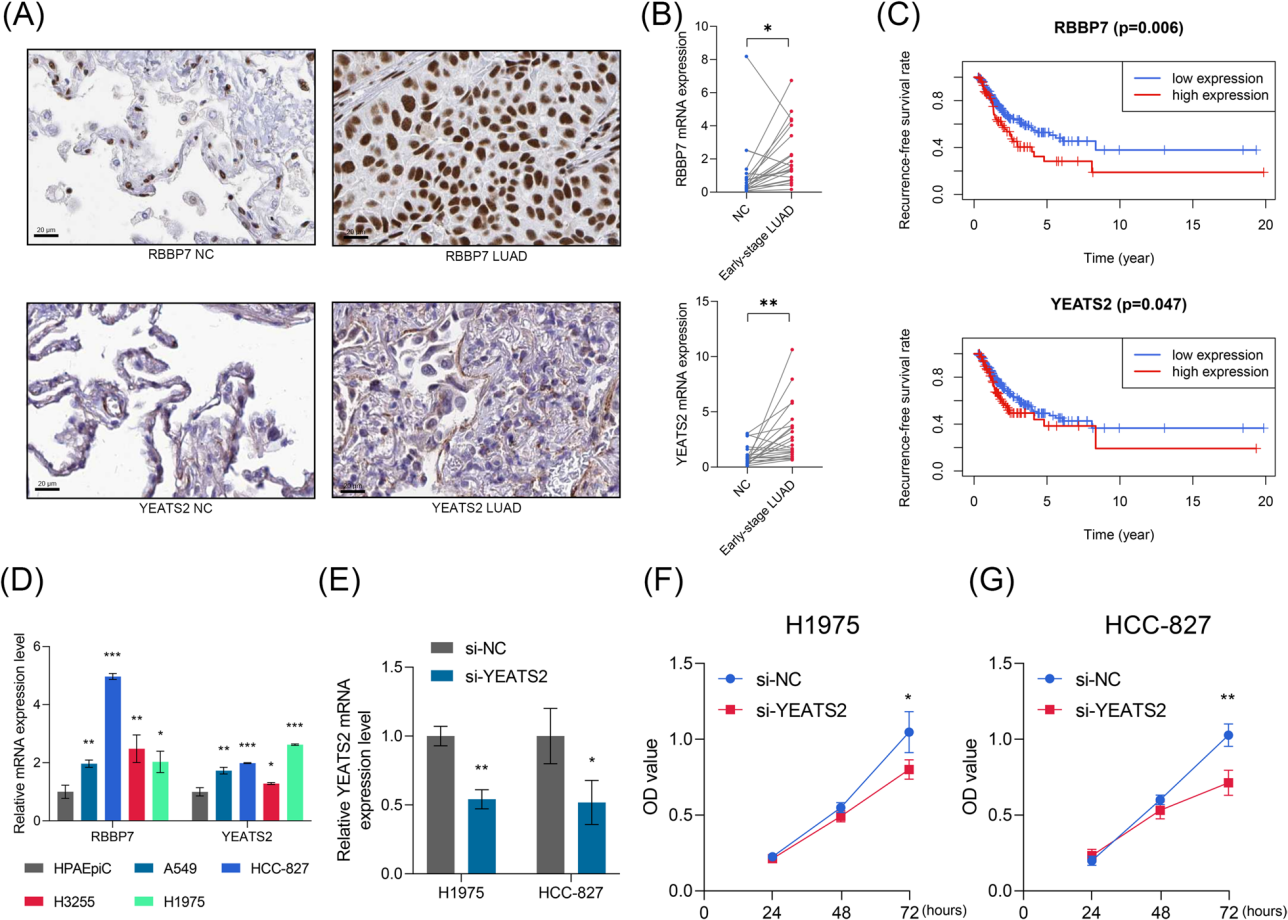
The external validation of RBBP7 and YEATS2. **A** The immunohistochemical staining showed that RBBP7 and YEATS2 were up-regulated in the LUAD samples compared with the normal lung tissue. **B** The RT-qPCR experiments showed that the expression levels of RBBP7 and YEATS2 in early-stage LUAD samples were higher than those in adjacent normal tissues in 23 clinical samples from the local hospital. **C** The predictive value of RBBP7 (up) and YEATS2 (down) to RFS in the TCGA cohort. The optimal cut-off was determined by the X-tile. **D** The expressions of RBBP7 and YEATS2 in human pulmonary alveolar epithelial cell line and LUAD cell lines, which were detected by RT-qPCR. **E** RT-qPCR experiments were carried out to verify the expression levels of YEATS2 in H1975 and HCC-827 cell lines transfected with si-NC or si-YEATS2. **F**–**G** The knockdown of YEATS2 significantly suppressed the proliferation of H1975 (**F**) and HCC-827 (**G**) cells. *LUAD* lung adenocarcinoma, *RFS* recurrence-free survival, *OD* optical density

Next, the expression level and the prognosis value of RBBP7 and YEATS2 were validated in external public dataset and the 23 local clinical samples. Compared with the normal lung tissue, the LUAD samples exhibited higher protein expression (Fig. 4A) and mRNA levels (Fig. 4B) of RBBP7 (P < 0.05) and YEATS2 (P < 0.01), which corresponds to the genomic difference analysis in the TCGA cohort (RBBP7, logFC = 14.460, FDR < 0.001; YEATS2, logFC = 3.189, FDR < 0.05). The higher expression levels of RBBP7 and YEATS2 heralded unfavorable prognosis in the TCGA cohort (all P < 0.05, Fig. 4C). Compared with human pulmonary alveolar epithelial cell, the LUAD cells exhibited higher levels of RBBP7 and YEATS2 (all P < 0.05, Fig. 4D). These evidences above suggested that both of RBBP7 and YEATS2 were oncogenes. Since the HR of YEATS2 was superior to that of RBBP7 in the univariate Cox regression analyses (Fig. 3F), we chose YEATS2 to conduct the experimental validation. Using the si-YEATS2, the YEATS2-knockdown cell lines were constructed (Fig. 4E). The CCK-8 assays indicated that the knockdown of YEATS2 significantly inhibited the proliferation of H1975 (P < 0.05, Fig. 4F) and HCC-827 (P < 0.01, Fig. 4G) cells. Pan-cancer analyses from the single-cell level indicated that RBBP7 and YEATS2 were both associated with multiple cancer functional statuses, re-confirming the vital roles of these genes in the initiation and progression of malignant cancers (Additional file 8: Fig. S2).

### Development and validation of an acetylation-related gene signature

**Fig. 5:**
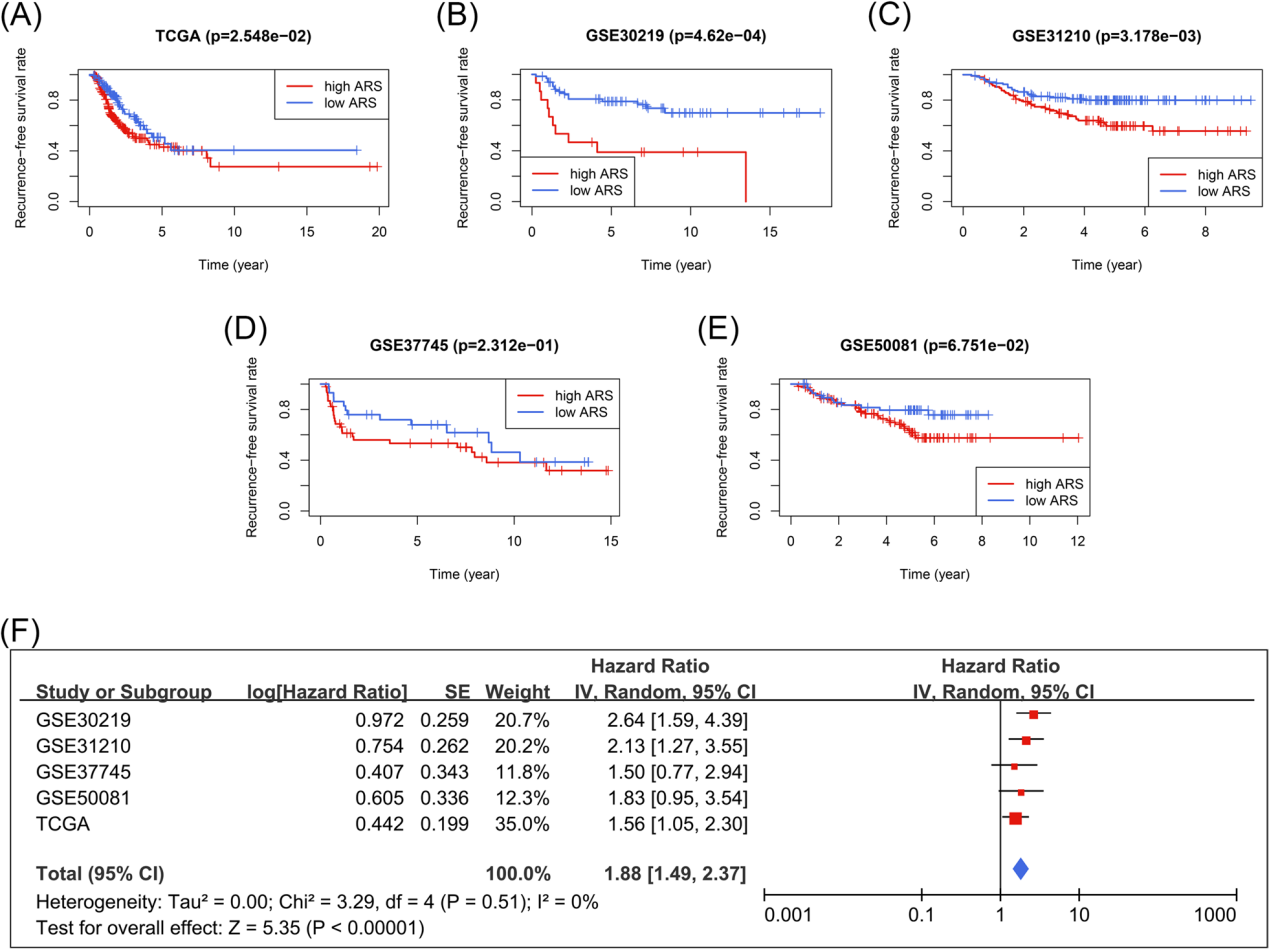
ARS was a robust biomarker for the RFS of early-stage LUAD patients. **A**–**E** The predictive performance of ARS in the TCGA cohort (**A**), GSE30219 cohort (**B**), GSE31210 cohort (**C**), GSE37745 cohort (**D**), and GSE50081 cohort (**E**). **F** The meta-analysis indicated that the early-stage LUAD patients with high ARS suffered poorer RFS. *LUAD* lung adenocarcinoma, *RFS* recurrence-free survival, *ARS* acetylation-related score, *TCGA* the Cancer Genome Atlas

RBBP7 and YEATS2 were ultimately included in the prognosis gene signature, and the predictive model was constructed as follows: $ARS=0.01288\times expression\left(YEATS2\right)+0.0059\times expression\left(RBBP7\right)$. According to the optimal cut-off value determined by X-tile, which equaled 0.9022, all the early-stage LUAD patients were divided into a high- or low-ARS subgroup. ARS was a significant biomarker to distinguish the low- and high-risk subjects in the TCGA cohort (n = 334, P < 0.05, Fig. 5A), GSE30219 cohort (n = 81, P < 0.001, Fig. 5B), and GSE31210 cohort (n = 226, P < 0.01, Fig. 5C). However, the statistical significance has not been observed in the GSE37745 cohort (n = 43, P > 0.05, Fig. 5D) and GSE50081 cohort (n = 121, P > 0.05, Fig. 5E). Subsequently, a meta-analysis was performed to clarify the prognosis value of ARS better. The pooled results indicated that ARS was a significant predictor of the RFS of early-stage LUAD patients (pooled HR = 1.88, 95% CI 1.49–2.37, P < 0.001, Fig. 5F), indicating the tremendous potential of ARS in clinical practice. The baseline clinical information of these cohorts was displayed in the previous study [23] and Additional file 9: Table S7.

### The clinical association of ARS

**Fig. 6:**
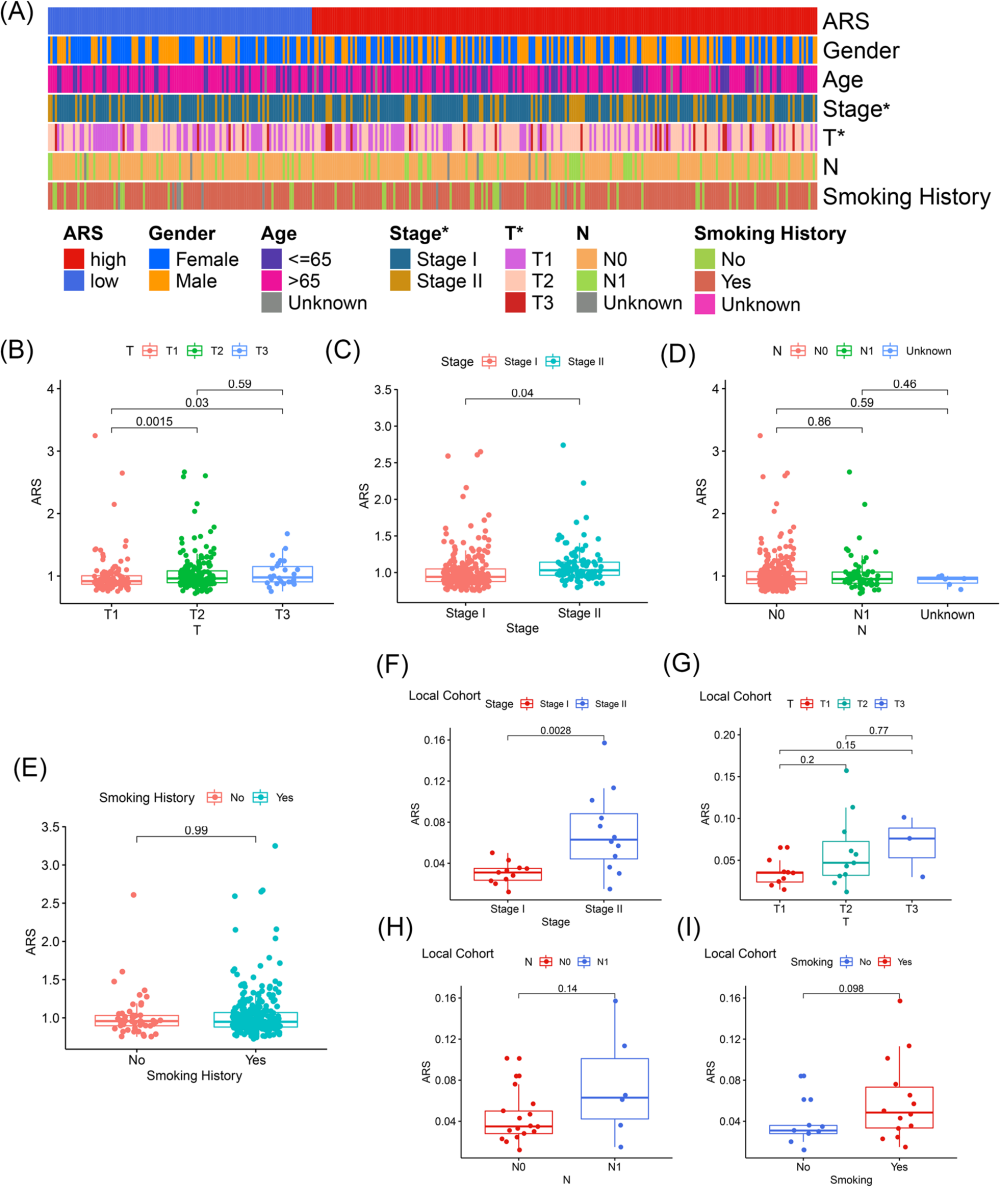
The clinicopathological association of ARS. **A** The heatmap indicated that ARS was significantly associated with pathological T stages and TNM stages of early-stage LUAD patients through the Chi-square test. **B**–**E** The Wilcoxon signed-rank tests displayed the association of ARS with pathological T stages (**B**), TNM stages (**C**), pathological N stages (**D**), and smoking history (**E**) in the TCGA cohort. The optimal cut-off of age is determined by the X-tile. **F**–**I** The Wilcoxon signed-rank tests indicated the association of ARS with TNM stages (**F**), pathological T stages (**G**), pathological N stages (**H**), and smoking history (**I**) in the local cohort. *LUAD* lung adenocarcinoma, *ARS* acetylation-related score; * *P* < 0.05

Pearson’s chi-square test indicated that ARS was significantly associated with pathological T stages (P < 0.05) and TNM stages (P < 0.05) in the early-stage LUAD patients from the TCGA (Fig. 6A). The difference of ARS exists primarily in T1 vs T2 (P < 0.01) and T1 vs T3 (P < 0.05, Fig. 6B). Additionally, we observed that ARS was positively associated with TNM stage in the TCGA-LUAD cohort (P < 0.05, Fig. 6C). Nevertheless, the significant association of ARS with N stages (P > 0.05, Fig. 6D) and smoking history (P > 0.05, Fig. 6E) has not been observed. In the local cohort, ARS was significantly associated with TNM stages (P < 0.01, Fig. 6F), but the non-significant association was observed in T stages (P > 0.05, Fig. 6G), N stages (P > 0.05, Fig. 6H), and smoking history (P > 0.05, Fig. 6I) possibly due to the relatively small-scale sample size. In addition, compared with these routine clinicopathological parameters, ARS was an independent prognosis factor no matter in the univariate or multivariate Cox analyses (P < 0.05, P < 0.05, Table 3).

**Table 3:** ARS is an independent predictor for the recurrence of early-stage LUAD

Parameters	Univariate Cox		Multivariate Cox	
	HR (95% CI)	P value	HR (95% CI)	P value
Gender (male vs. female)	1.045 (0.676–1.616)	0.842	1.128 (0.720–1.769)	0.599
Age (< = 65 vs. > 65)	1.203 (0.742–1.951)	0.453	1.291 (0.793–2.103)	0.305
Stage (stage I vs. stage II)	1.814 (1.165–2.825)	0.008	1.884 (0.669–5.302)	0.230
T (T1–2 vs. T3)	4.018 (2.051–7.870)	< 0.001	2.457 (0.766–7.878)	0.131
N (N0 vs. N1)	1.174 (0.703–1.962)	0.540	0.728 (0.244–2.171)	0.569
Smoking (no vs. yes)	1.178 (0.650–2.134)	0.589	1.384 (0.747–2.563)	0.301
ARS (low vs. high)	1.341 (1.044–2.129)	0.039	1.291 (1.007–2.069)	0.044

*ARS* acelylation-related score, *HR* hazard ratio, *CI* confidence interval, *LUAD* lung adenocarcinoma

### Construction of a prognosis nomogram including ARS

**Fig. 7:**
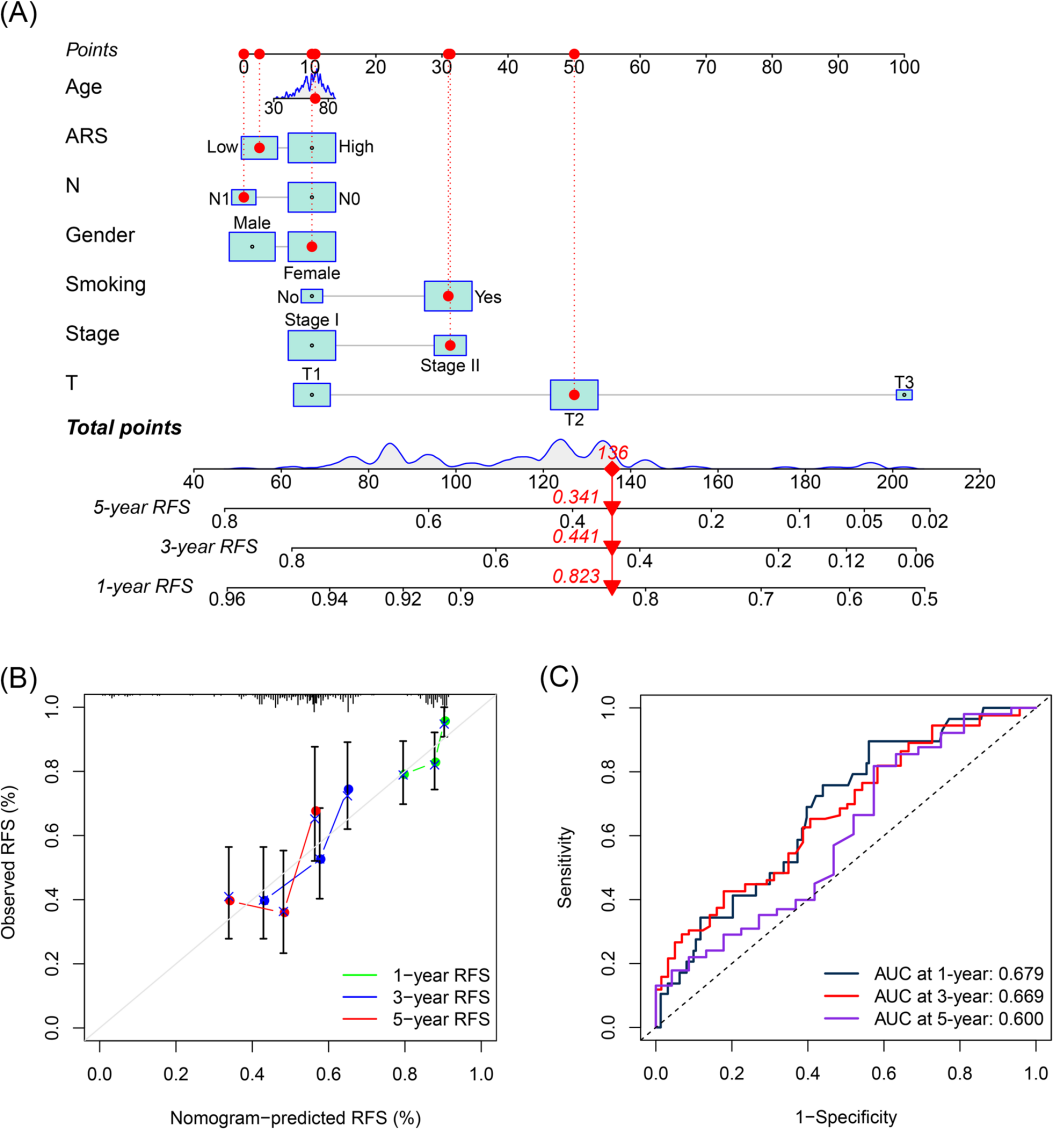
The development of validation of a nomogram. **A** The established nomogram including ARS and clinicopathological features. **B** The calibration curve of the nomogram. **C** The time-dependent ROC analyses of the nomogram. *ARS* acetylation-related score, *ROC* receiver operating curve

To achieve a better predictive performance, a nomogram, including ARS and other clinical features, was developed (Fig. 7A). The calibration curves for the recurrence statuses at 1, 3, and 5 years indicated that the nomogram could predict the RFS with high efficacy (Fig. 7B). The time-dependent ROC analysis showed that the areas under curves (AUCs) of ARS were 0.679, 0.669, and 0.600 for 1-, 3-, and 5-year RFS (Fig. 7C).

### Single-cell sequencing analyses

**Fig. 8:**
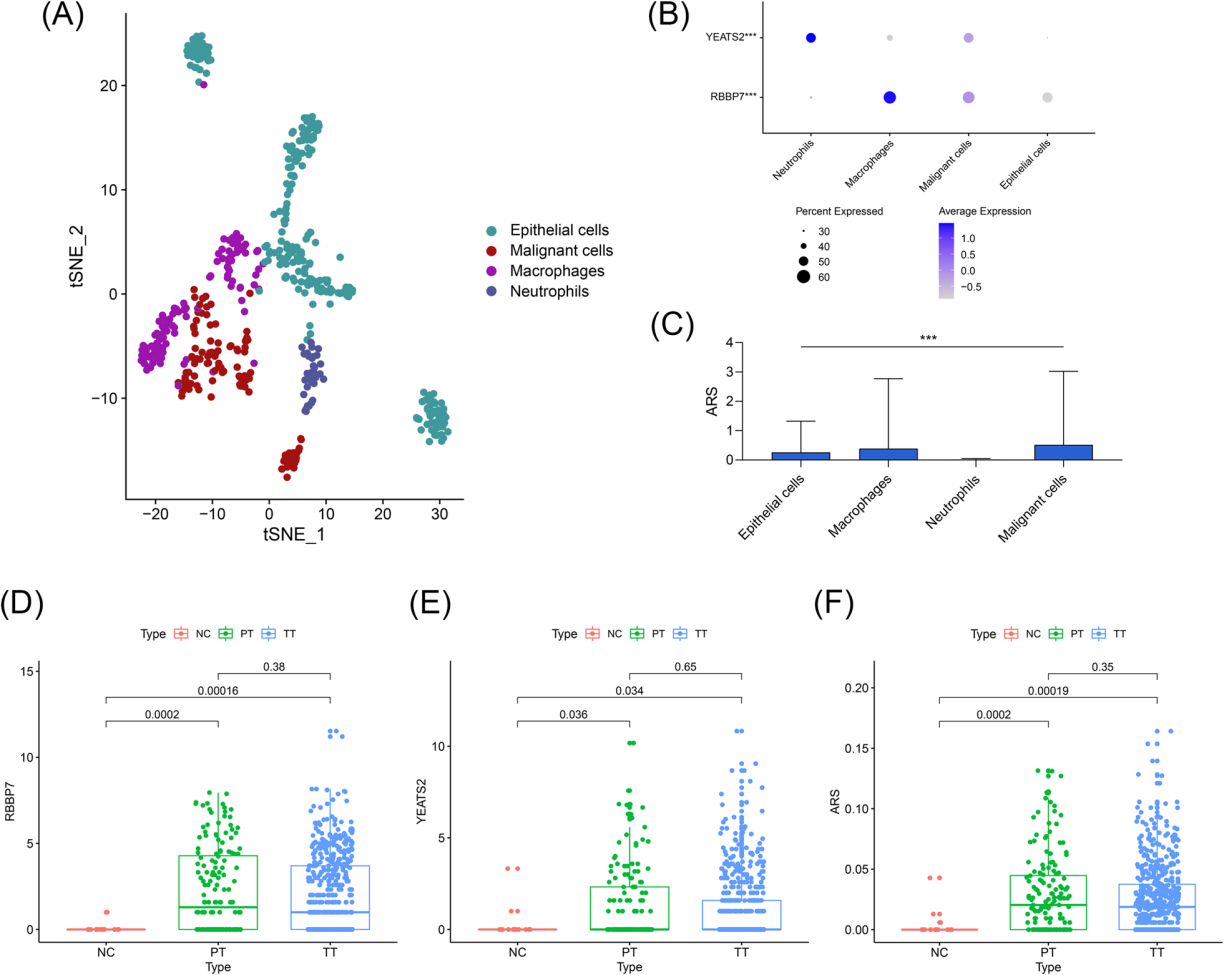
The single-cell RNA sequencing analysis of the early-stage LUAD patients. **A** 520 cell samples isolated TT were clustered and annotated with four cell types. **B** YEATS2 and RBBP7 were significantly up-regulated in the neutrophils and macrophages, respectively, which was detected by the Kruskal–Wallis test. **C** The levels of ARS in each cell type. **D**–**F** The levels of RBBP7 (**D**), YEATS2 (**E**), and ARS (**F**) in the cells from the NC, PT, and TT samples. *LUAD* lung adenocarcinoma, *TT* tumor tissue, *PT* paratumor tissue, *NC* negative control, *ARS* acetylation-related score; ***P < 0.001

After the quality control, all the 520 cell samples from TT were included in the further study. 520 cells were clustered into nine cell groups (Additional file 10: Fig. S3A), and their marker genes’ expression levels in each cell are shown in Additional file 10: Fig. S3B. 9 cell clusters were mainly annotated with four cell types, including epithelial cells, malignant cells, macrophages, and neutrophils (Fig. 8A). Interestingly, we observed that YEATS2 was primarily expressed on neutrophils (P < 0.001, Fig. 8B) and RBBP7 was predominantly expressed in macrophages (P < 0.001, Fig. 8B), implying both of these genes are involved in tumor immune response of early-stage LUAD. ARS, which was based on the expression value of RBBP7 and YEATS2, was also significantly altered among these cell types (P < 0.001, Fig. 8C). Additionally, the expression of RBBP7 and YEATS2 and ARS in the NC, PT, and TT samples were also detected, as shown in Fig. 8D–F, respectively. The levels of RBBP7, YEATS2, and ARS were significantly evaluated in the TT and PT samples compared with the NC samples (all P < 0.05), but no statistical significance was found between the PT and TT samples (all P > 0.05), indicating that ARS was possibly associated with tumor immunity of early-stage LUAD.

### ARS is a potential biomarker for immunotherapeutic effectiveness

**Fig. 9:**
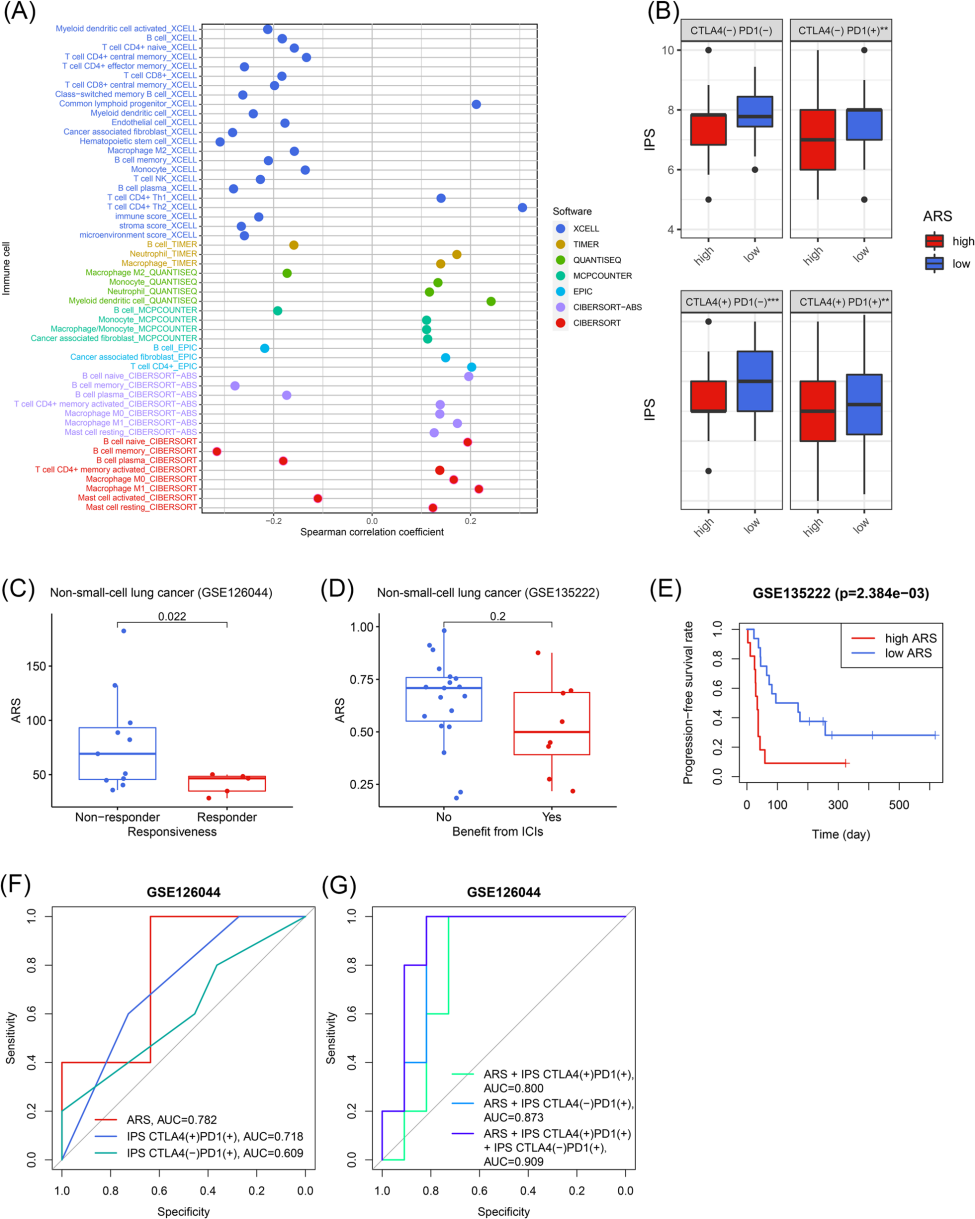
ARS served as a promising predictor for immunotherapeutic efficacy. **A** The association between ARS and the immune cell infiltration levels in the early-stage LUAD subjects from TCGA. **B** ARS was negatively associated with IPS. **C** and **D** The ARS levels among the responders and non-responders to ICIs in the GSE126044 cohort (**C**) and GSE135222 cohort (**D**). **E** ARS could distinguish the subjects with poor progression-free survival rates in the GSE135222 cohort. **F** ARS was superior to IPS to predict the anti-PD-1 treatment effectiveness in the GSE126044 cohort. **G** The combination of ARS and IPS could better evaluate the immunotherapeutic efficacy in the GSE126044 cohort. *ARS* acetylation-related score, *LUAD* lung adenocarcinoma, *TCGA* the Cancer Genome Atlas, *IPS* immunophenoscore, *ICIs* immune checkpoint inhibitors; **P < 0.01; ***P < 0.001

Given that RBBP7 and YEATS2 were both associated with the immune response of the early-stage LUAD samples, we then explored the efficacy of ARS to predict the immunotherapeutic outcomes. First, the correlation between ARS and multiple immune cell infiltration levels was calculated (Fig. 9A). We found that ARS was obviously associated with multiple important immune cell infiltration in the early-stage LUAD samples, such as B cells (negatively associated), neutrophils (positively associated) and macrophages (positively associated). It was reported that B cells could promote immunotherapy response [24], neutrophils could inhibit the cytotoxicity of T cells [25], and macrophages were able to perform the immuno-suppressive function in malignant cancers [26], implying that the patients with low ARS were more likely to benefit from immunotherapy. Additionally, it was observed that the high-ARS patients with early-stage LUAD exhibited lower IPS, re-confirming the predictive potential of ARS (all P < 0.05, Fig. 9B). Next, the cohorts receiving immunotherapy were used to better clarify the clinical usefulness. In the GSE126044 cohort (n = 16), the responders to ICIs showed significantly lower ARSs compared with the non-responders (P < 0.05, Fig. 9C). In the GSE13522 cohort (n = 27), though the difference in significance of ARS between the responders and non-responders has not been observed (P > 0.05, Fig. 9D), the subjects with high ARS suffered poorer progression-free survival (P < 0.01, Fig. 9E). In addition, the comparison of the predictive performance to immunotherapeutic effectiveness of ARS and IPS was also conducted in the GSE126044 cohort. Compared with IPS, ARS exhibited a stronger predictive ability to the anti-PD-1 treatment outcomes (AUC = 0.782, Fig. 9F), and the combination of IPS and ARS seemed a more powerful predictive biomarker (AUC = 0.909, Fig. 9G). The IPS of each patient in the GSE126044 cohort was obtained from TCIA after uploading the transcriptome sequencing data to this website. To sum up, ARS was a promising clinical tool to evaluate the immunotherapeutic response of early-stage LUAD.

## Discussion

As one of the most common forms of epigenetic modification, acetylation refers to the process that the protein or mRNA is grafted with acetyl groups. Most current researches focus on histone acetylating modification, which can influence apoptosis, necrosis, autophagy, reactive oxygen species level, angiogenesis, and immunomodulatory effects of tumor cells [27]. However, a recent study found that acetylation could also occur in mRNA and affect the mRNA stability and translation efficiency, indicating the vital role of acetylation in cell life activities [6]. To date, some critical genes associated with acetylation have been reported in LUAD, such as ESCO2 [28], THAP7 [19], and CBP [20], deepening our understanding of its pathological mechanisms. The non-negligible influence of acetylation on tumor immunity has also been reported, and many genes belonging to the histone acetyltransferase family or the histone deacetylase family also serve as tumor immunity regulators, such as GCN5, p300, Tip60, HAT1, and HADCs [29]. However, the correlation between acetylation and immunotherapy efficacy remains unclear [29]. Additionally, although the prognosis of early-stage LUAD patients is much better than that of advanced LUAD, the recurrence rate of early-stage is about 30–45%, as stated above, and more precisions for the recurrence of early-stage LUAD are urgently demanded. Nevertheless, to the best of our knowledge, the hub acetylation-related genes as the recurrence and immunotherapeutic effectiveness biomarkers in early-stage LUAD have not been systematically analyzed.

First, the present study collected the acetylation-related genes from the public database and conducted the unsupervised clustering based on the TCGA. It was found that the acetylation-based clustering was significantly associated with the recurrence and tumor immune response of early-stage LUAD patients, implying that acetylation might play a vital role in the pathological process of early-stage LUAD. After the systematical retrieval of GEO datasets, GSE30219, GSE31210, GSE37745, and GSE50081 were downloaded for the validation of the established model’s predictive performance to RFS; meanwhile, GSE135222, GSE126044, and GSE198291 was used to verify the association of the predictive model with tumor immune response and immunotherapeutic efficacy. Next, through a series of bioinformatical and dimension-reduction methods, including genomic divergence analysis, PPI network construction, Lasso regression, univariate Cox regression, and multivariate Cox regression, a two-gene signature containing RBBP7 and YEATS2 was developed, according to which, the ARS of each subject enrolled was calculated. The meta-analysis results revealed that ARS was a reliable predictor for the RFS of early-stage LUAD patients. The Rt-qPCR experiments of the 23 clinical samples from the local hospital indicated that the ARS was significantly associated with risk clinical parameters. The in vitro cell experiments indicated that the knock-down of YEATS2 obviously suppressed the proliferation of H1975 and HCC-827 cells. Compared with the routine clinicopathological features, ARS was an independent prognosis factor for the RFS of early-stage LUAD patients. A nomogram was constructed to boost the predictive performance even further. Last, the scRNA-seq analyses and the cohort studies in which the subjects have received ICIs treatment indicated that ARS was a promising clinical tool to evaluate the immunotherapeutic effect.

The rapid development of genomic sequencing technology attenuates the cost of tumor genetic testing and spawns the construction of gene panels useful for diagnosis, prognosis, or medical treatment sensitivity evaluation [30–33]. Here, the established parsimonious model only contained RBBP7 and YEATS2, possibly further reducing the testing cost in clinical practice. RBBP7, a ubiquitously expressed nuclear protein, is extensively involved in diverse biological processes, such as oocyte maturation [34], decidualization [35], and mitochondrial biogenesis and function maintaining [36], by enhancing protein deacetylation. Previous studies reported that RBBP7 was capable of promoting tumor cell proliferation, migration, invasion, and glycolysis and served as a prognosis predictor in esophageal cancer [37, 38], but the roles of RBBP7 in lung cancer, especially in early-stage LUAD, are still unclear. In this study, we found that RBBP7 could act as a predictor for the recurrence of early-stage LUAD and is mainly expressed in macrophages, implying that RBBP7 might affect the progression of LUAD through interacting macrophages. YEATS2 is a histone H3K27 acetylation reader, which can bind to acetylated histone H3 through the YEATS domain, and it was considered a connector between histone acetylation and lung cancer [39]. The high expression of YEATS2 heralded the poor prognosis of lung cancer patients, which is consistent with our findings [39]. However, how YEATS2 is involved in the malignant phenotypes of lung cancer has not been fully clarified. Here, the present study disclosed that YEATS2 was highly expressed on neutrophils in the tumor microenvironment, which provided a novel cut-in point to elucidate the biological functions of YEATS2 in tumorigenesis.

However, some shortcomings of this study should be acknowledged. First, despite the fact that this is a comprehensive study and the conclusions have been confirmed in multiple independent cohorts and local clinical samples, a prospective, multi-center, large-scale clinical trait would be more beneficial to clarify the usefulness of ARS. Second, the biological functions of RBBP7 and YEATS2, especially in the macrophages and neutrophils, are obscure, and in vivo and in vitro experiments are needed for further study.

## Conclusion

In conclusion, a novel gene signature containing RBBP7 and YEATS2 was developed and externally validated to evaluate the recurrence and immunotherapeutic efficacy of early-stage LUAD patients, providing the possible cut-in points in the molecular mechanism exploration and a promising clinical tool to guide the personalized treatment.

## Supplementary Information

**Additional file 1. Table S1**: The acetylation-related genes collected from the MSigDB.

**Additional file 2. Table S2**: The information of the clinical samples obtained from the local hospital.

**Additional file 3. Table S3**: The clustering statuses of the early-stage LUAD patients from TCGA.

**Additional file 4. Fig. S1**: The GSEA indicated that the early-stage LUAD patients in C2 subgroup exhibited stronger immune response.

**Additional file 5. Table S4**: The comparison of the immune cell infiltration proportion between C1 and C2 subgroups.

**Additional file 6. Table S5**: The importance of genes in the PPI network.

**Additional file 7. Table S6**: The univariate Cox regression of the Top 30 genes showing the highest degree.

**Additional file 8. Fig. S2**: The pan-cancer analyses of RBBP7 (A) and YEATS2 (B) from the single-cell level.

**Additional file 9. Table S7**: The baseline clinicopathological information of the training and external validation cohorts.

**Additional file 10. Fig. S3**: The process of single-cell RNA sequencing analyses. A 520 cell samples from tumor tissue were divided into 9 cell clusters. B The expression level of the marker genes in each cluster. The colors ranging from purple to yellow represented the expression values from low to high.

## Data Availability

The transcription sequencing data and corresponding clinical information of TCGA-LUAD cohort are available from TCGA (https://portal.gdc.cancer.gov/). GSE30219 (https://www.ncbi.nlm.nih.gov/geo/query/acc.cgi?acc=GSE30219), GSE31210 (https://www.ncbi.nlm.nih.gov/geo/query/acc.cgi?acc=GSE31210), GSE37745 (https://www.ncbi.nlm.nih.gov/geo/query/acc.cgi?acc=GSE37745), GSE50081 (https://www.ncbi.nlm.nih.gov/geo/query/acc.cgi?acc=GSE50081), GSE198291 (https://www.ncbi.nlm.nih.gov/geo/query/acc.cgi?acc=GSE198291), GSE135222 (https://www.ncbi.nlm.nih.gov/geo/query/acc.cgi?acc=GSE135222), and GSE126044 (https://www.ncbi.nlm.nih.gov/geo/query/acc.cgi?acc=GSE126044) datasets are obtained from GEO (https://www.ncbi.nlm.nih.gov/geo/). 240 acetylation-related genes are collected from MSigDB (https://www.gsea-msigdb.org/gsea/msigdb/). The immunophenoscore of each LUAD patient from TCGA is available from TCIA (https://tcia.at/home). The marker genes of the cells in tumor microenvironment are obtained from CellMarker (http://bio-bigdata.hrbmu.edu.cn/CellMarker/) and CancerSEA (http://biocc.hrbmu.edu.cn/CancerSEA/). STRING (https://cn.string-db.org/) is used to detect the interaction relationship of the genes, and the Human Protein Atlas (https://www.proteinatlas.org/) is used to detect the protein expression level of the genes. All the data deposited in the databases mentioned above is publicly available. The code would be supplied from the corresponding author upon request.

## Abbreviations

LUAD: Lung adenocarcinoma
ICI: Immune checkpoint inhibitor
MSigDB: Molecular Signatures Database
TCGA: The Cancer Genome Atlas
GEO: Gene Expression Omnibus
RFS: Recurrence-free survival
scRNA-seq: Single-cell RNA sequencing
RNA-seq: RNA-sequencing
TPM: Transcripts per kilobase of exon model per million mapped reads
TNM: Tumor-node-metastasis
RT-qPCR: Real-time quantitative PCR
CCK-8: Cell counting kit-8
OD: Optical density
TT: Tumor tissue
PT: Paratumor tissue
NC: Negative control
PCA: Principal component analysis
PC: Principal component
t-SNE: T-distributed stochastic neighbor embedding
CDF: Cumulative distribution function
GSEA: Gene Set Enrichment Analysis
NES: Normalized enrichment score
FDR: False discovery rate
DEG: Differentially-expressed gene
PPI: Protein–protein interaction
Lasso: Least absolute shrinkage and selection operator
HR: Hazard ratio
CI: Confidence interval
ARS: Acetylation-related score
ROC: Receiver operating curve
IPS: Immunophenoscore
TCIA: The Cancer Immunome Atlas
RBBP7: RB Binding Protein 7, Chromatin Remodeling Factor
YEATS2: YEATS Domain Containing 2
AUC: Area under curve
