# Supplementary material for: Construction and experimental validation of an acetylation-related gene signature to evaluate the recurrence and immunotherapeutic response in early-stage lung adenocarcinoma
Source: BMC Med Genomics. 2022 Dec 11;15:254. doi: 10.1186/s12920-022-01413-7 (PMC9741798; doi:10.1186/s12920-022-01413-7)
Supplement: Supplementary file 1 — Additional file 1. Table S1: The acetylation-related genes collected from the MSigDB. [file 12920_2022_1413_MOESM1_ESM.docx]

**Additional file 1: Table S1** The acetylation-related genes collected from the MSigDB.

| Name | Description | Genes |
| --- | --- | --- |
| BIOCARTA_RELA_PATHWAY | Acetylation and Deacetylation of RelA in The Nucleus | CHUK, CREBBP, EP300, FADD, IKBKB, IKBKG, NFKB1, NFKBIA, RELA, RIPK1, TNF, TNFRSF1A, TNFRSF1B, TRADD, TRAF6. |
| GOBP_N_TERMINAL_PEPTIDYL_METHIONINE_ACETYLATION | The acetylation of the N-terminal methionine of proteins to form the derivative N-acetyl-L-methionine. [RESID:AA0049] | NAA15, NAA16, NAA20, NAA25, NAA30, NAA35, NAA60. |
| GOBP_N_TERMINAL_PROTEIN_AMINO_ACID_ACETYLATION | The acetylation of the N-terminal amino acid of proteins. [GOC:ai] | AANAT, CREBBP, EP300, KAT2B, NAA10, NAA11, NAA15, NAA16, NAA20, NAA25, NAA30, NAA35, NAA40, NAA50, NAA60, NAA80, SOX4. |
| GOBP_PEPTIDYL_LYSINE_ACETYLATION | The acetylation of peptidyl-lysine. [GOC:mah] | ACTB, ACTL6A, ACTL6B, APBB1, ARID5A, ARRB1, ATAT1, ATF2, ATG5, ATXN7, ATXN7L3, AUTS2, BAG6, BEND3, BLOC1S1, BRCA1, BRCA2, BRD1, BRD7, BRD8, BRPF1, BRPF3, CAMK1, CDY1, CDY1B, CDY2A, CDY2B, CHD5, CHEK1, CLOCK, CPA4, CREBBP, CRTC2, CTBP1, CTCF, DIP2A, DIP2B, DMAP1, DR1, EID1, ENY2, EP300, EP400, EPC1, EPC2, ESCO1, FLCN, FOXP3, GATA2, GATA3, GLYR1, GTF2B, GTF3C4, HAT1, HCFC1, HDAC2, HDAC5, HINT2, IL1B, ING3, ING4, ING5, IRF4, ISL1, IWS1, JADE1, JADE2, JADE3, KANSL1, KANSL2, KANSL3, KAT14, KAT2A, KAT2B, KAT5, KAT6A, KAT6B, KAT7, KAT8, KLF15, KMT2A, LDB1, LEF1, LIF, MAP3K7, MAPK3, MBD3, MBIP, MBTD1, MCM3AP, MCRS1, MEAF6, MECP2, MED24, MLLT1, MLLT3, MORF4L1, MORF4L2, MRGBP, MSL1, MSL2, MSL3, MSL3P1, MUC1, MYOCD, MYOD1, NAA40, NAA50, NAA60, NAP1L2, NAT8, NAT8B, NCOA1, NCOA3, NFYA, NFYB, NFYC, NOC2L, NOS1, OGT, PARK7, PAXIP1, PBXIP1, PCGF2, PER1, PHF20, PHF20L1, PIH1D1, PIWIL2, PML, POLE3, POR, PPARGC1A, PRKAA1, PRKAA2, PYGO2, RAPGEF3, RPS6KA4, RPS6KA5, RUVBL1, RUVBL2, SDR16C5, SET, SETD5, SGF29, SIN3A, SIRT1, SMAD4, SMARCB1, SMC5, SNAI2, SNCA, SOX4, SPHK2, SPI1, SRCAP, SUPT3H, SUPT7L, TADA1, TADA2A, TADA2B, TADA3, TAF1, TAF10, TAF12, TAF1L, TAF2, TAF4, TAF5, TAF5L, TAF6, TAF6L, TAF7, TAF9, THAP7, TRIM16, TRRAP, TWIST1, USP22, VPS72, WBP2, WDR5, YEATS2, YEATS4, ZMPSTE24, ZNF451, ZZZ3. |
| GOCC_H3_HISTONE_ACETYLTRANSFERASE_COMPLEX | A multisubunit complex that catalyzes the acetylation of histone H3. [GOC:mah] | BRD1, BRPF1, BRPF3, ING4, ING5, KAT6A, KAT6B, KAT7, MEAF6, PHF14. |
| GOCC_H4_H2A_HISTONE_ACETYLTRANSFERASE_COMPLEX | A multisubunit complex that catalyzes the acetylation of histones H4 and H2A. [GOC:mah, GOC:rb] | ACTB, ACTBL2, ACTL6A, ACTL6B, BRD8, DMAP1, EP400, EPC1, EPC2, ING3, KAT5, MBTD1, MEAF6, MLLT1, MLLT3, MORF4L1, MORF4L2, MRGBP, MSL3, MSL3P1, RUVBL1, RUVBL2, TRRAP, VPS72, YEATS2, YEATS4. |
| GOCC_MSL_COMPLEX | A histone acetyltransferase complex that catalyzes the acetylation of a histone H4 lysine residue at position 16. In human, it contains the catalytic subunit MOF, and MSL1, MSL2 and MSL3. [PMID:16227571, PMID:20018852] | KAT8, MSL1, MSL2, MSL3, MSL3P1. |
| GOCC_NSL_COMPLEX | A histone acetyltransferase complex that catalyzes the acetylation of a histone H4 lysine residues at several positions. In human, it contains the catalytic subunit MOF, NSL1/KIAA1267, NSL2/KANSL2, NSL3/KANSL3, MCRS1, PHF20, OGT1, WDR5 and HCF1. [GOC:lb, PMID:20018852] | HCFC1, KANSL1, KANSL1L, KANSL2, KANSL3, KAT8, MCRS1, OGT, PHF20, PHF20L1, WDR5. |
| GOMF_PEPTIDE_ALPHA_N_ACETYLTRANSFERASE_ACTIVITY | Catalysis of the reaction: acetyl-CoA + peptide = CoA + N-alpha-acetylpeptide. This reaction is the acetylation of the N-terminal amino acid residue of a peptide or protein. [GOC:mah, PMID:30054468] | NAA10, NAA11, NAA15, NAA16, NAA20, NAA25, NAA30, NAA40, NAA50, NAA60, NAA80. |
| GOMF_PEPTIDE_N_ACETYLTRANSFERASE_ACTIVITY | Catalysis of the acetylation of an amino acid residue of a peptide or protein, according to the reaction: acetyl-CoA + peptide = CoA + N-acetylpeptide. [GOC:mah] | ARRB1, ATAT1, ATF2, BAZ1A, BRCA2, CDY1, CDY1B, CDY2A, CDY2B, CLOCK, CREBBP, EP300, ESCO1, GTF2B, GTF3C4, HAT1, KAT14, KAT2A, KAT2B, KAT5, KAT6A, KAT6B, KAT7, KAT8, MCM3AP, MED24, MSL3, NAA10, NAA11, NAA15, NAA16, NAA20, NAA25, NAA30, NAA40, NAA50, NAA60, NAA80, NCOA1, NCOA3, SRCAP, SUPT7L, TADA2A, TAF1, TAF10, TAF1L, TAF9, USP22. |
| REACTOME_PI5P_REGULATES_TP53_ACETYLATION | PI5P Regulates TP53 Acetylation | EP300, ING2, MAP2K6, PIN1, PIP4K2A, PIP4K2B, PIP4K2C, PIP4P1, TP53. |
| REACTOME_REGULATION_OF_FOXO_TRANSCRIPTIONAL_ACTIVITY_BY_ACETYLATION | Regulation of FOXO transcriptional activity by acetylation | CREBBP, EP300, FOXO1, FOXO3, FOXO4, KAT2B, SIRT1, SIRT3, TXN, TXNIP. |
| REACTOME_REGULATION_OF_TP53_ACTIVITY_THROUGH_ACETYLATION | Regulation of TP53 Activity through Acetylation | AKT1, AKT2, AKT3, BRD1, BRD7, BRPF1, BRPF3, CHD3, CHD4, EP300, GATAD2A, GATAD2B, HDAC1, HDAC2, ING2, ING5, KAT6A, MAP2K6, MBD3, MEAF6, MTA2, PIN1, PIP4K2A, PIP4K2B, PIP4K2C, PIP4P1, PML, RBBP4, RBBP7, TP53. |
| GOCC_ATAC_COMPLEX | A chromatin remodelling complex that regulates transcription via acetylation primarily of nucleosomal histones H3 and possibly H4. Shares the histone acetylation (HAT) module of GCN5/PCAF-ADA2-ADA3-SGF29 (or orthologs) with the related SAGA complex (GO:0000124). Contains HAT subunits GCN5 or PCAF in a mutually exclusive manner. In addition to the HAT module contains DR1/NC2B, KAT14, MBIP, WDR5, YEATS2 and ZZZ3 or orthologs. Also regulates the activity of non-histone targets and orchestrates mitotic progression by regulating Cyclin A degradation through acetylation. [GOC:bhm, PMID:19936620, PMID:20562830, PMID:28966424] | DR1, KAT14, KAT2A, KAT2B, MAP3K7, MBIP, POLE3, POLE4, SGF29, TADA2A, TADA3, WDR5, YEATS2, ZZZ3. |

*MSigDB, Molecular Signatures Database.*
