# Supplementary material for: Construction and experimental validation of an acetylation-related gene signature to evaluate the recurrence and immunotherapeutic response in early-stage lung adenocarcinoma
Source: BMC Med Genomics. 2022 Dec 11;15:254. doi: 10.1186/s12920-022-01413-7 (PMC9741798; doi:10.1186/s12920-022-01413-7)
Supplement: Supplementary file 2 — Additional file 2. Table S2: The information of the clinical samples obtained from the local hospital. [file 12920_2022_1413_MOESM2_ESM.docx]

**Additional file 2: Table S2** The information of the clinical samples obtained from the local hospital.

| Sample ID | Group | Smoking | Stage | T | N | RBBP7_mRNA_expression | YEATS2_mRNA_expression | ARS |
| --- | --- | --- | --- | --- | --- | --- | --- | --- |
| LUAD1 | LUAD | Yes | Stage I | T1 | N0 | 0.901 | 1.429 | 0.024 |
| LUAD2 | LUAD | Yes | Stage I | T2 | N0 | 0.429 | 1.559 | 0.023 |
| LUAD3 | LUAD | Yes | Stage II | T3 | N0 | 2.262 | 1.312 | 0.030 |
| LUAD4 | LUAD | No | Stage I | T2 | N0 | 0.552 | 0.662 | 0.012 |
| LUAD5 | LUAD | No | Stage II | T2 | N0 | 1.274 | 5.962 | 0.084 |
| LUAD6 | LUAD | Yes | Stage II | T2 | N1 | 1.857 | 7.951 | 0.113 |
| LUAD7 | LUAD | Yes | Stage I | T2 | N0 | 4.036 | 0.725 | 0.033 |
| LUAD8 | LUAD | No | Stage II | T1 | N1 | 1.593 | 4.327 | 0.065 |
| LUAD9 | LUAD | Yes | Stage II | T3 | N0 | 4.403 | 5.798 | 0.101 |
| LUAD10 | LUAD | Yes | Stage II | T2 | N1 | 3.404 | 10.632 | 0.157 |
| LUAD11 | LUAD | Yes | Stage I | T1 | N0 | 1.586 | 1.998 | 0.035 |
| LUAD12 | LUAD | Yes | Stage I | T1 | N0 | 1.282 | 0.953 | 0.020 |
| LUAD13 | LUAD | Yes | Stage II | T2 | N1 | 2.212 | 3.729 | 0.061 |
| LUAD14 | LUAD | No | Stage I | T2 | N0 | 1.340 | 2.688 | 0.043 |
| LUAD15 | LUAD | No | Stage II | T3 | N0 | 4.875 | 3.682 | 0.076 |
| LUAD16 | LUAD | No | Stage II | T1 | N1 | 4.315 | 0.815 | 0.036 |
| LUAD17 | LUAD | No | Stage II | T1 | N1 | 0.751 | 0.821 | 0.015 |
| LUAD18 | LUAD | No | Stage I | T1 | N0 | 1.414 | 3.211 | 0.050 |
| LUAD19 | LUAD | No | Stage II | T2 | N0 | 0.162 | 3.556 | 0.047 |
| LUAD20 | LUAD | Yes | Stage II | T2 | N0 | 6.735 | 1.367 | 0.057 |
| LUAD21 | LUAD | Yes | Stage I | T1 | N0 | 2.201 | 1.146 | 0.028 |
| LUAD22 | LUAD | Yes | Stage I | T1 | N0 | 0.701 | 2.431 | 0.035 |
| LUAD23 | LUAD | Yes | Stage I | T2 | N0 | 1.623 | 1.672 | 0.031 |
| NC1 | NC | / | / | / | / | 1.126 | 1.619 | / |
| NC2 | NC | / | / | / | / | 0.310 | 1.835 | / |
| NC3 | NC | / | / | / | / | 0.558 | 0.772 | / |
| NC4 | NC | / | / | / | / | 0.079 | 0.128 | / |
| NC5 | NC | / | / | / | / | 0.137 | 0.521 | / |
| NC6 | NC | / | / | / | / | 0.401 | 0.947 | / |
| NC7 | NC | / | / | / | / | 0.596 | 0.584 | / |
| NC8 | NC | / | / | / | / | 2.524 | 0.848 | / |
| NC9 | NC | / | / | / | / | 8.190 | 3.043 | / |
| NC10 | NC | / | / | / | / | 0.801 | 0.379 | / |
| NC11 | NC | / | / | / | / | 2.548 | 0.747 | / |
| NC12 | NC | / | / | / | / | 0.732 | 0.831 | / |
| NC13 | NC | / | / | / | / | 0.274 | 2.836 | / |
| NC14 | NC | / | / | / | / | 0.744 | 0.585 | / |
| NC15 | NC | / | / | / | / | 0.218 | 0.250 | / |
| NC16 | NC | / | / | / | / | 0.885 | 0.322 | / |
| NC17 | NC | / | / | / | / | 0.200 | 0.849 | / |
| NC18 | NC | / | / | / | / | 0.443 | 0.948 | / |
| NC19 | NC | / | / | / | / | 0.063 | 0.216 | / |
| NC20 | NC | / | / | / | / | 1.387 | 1.109 | / |
| NC21 | NC | / | / | / | / | 0.388 | 0.278 | / |
| NC22 | NC | / | / | / | / | 0.194 | 0.409 | / |
| NC23 | NC | / | / | / | / | 0.202 | 2.943 | / |
