# Supplementary material for: Construction and experimental validation of an acetylation-related gene signature to evaluate the recurrence and immunotherapeutic response in early-stage lung adenocarcinoma
Source: BMC Med Genomics. 2022 Dec 11;15:254. doi: 10.1186/s12920-022-01413-7 (PMC9741798; doi:10.1186/s12920-022-01413-7)
Supplement: Supplementary file 3 — Additional file 3. Table S3: The clustering statuses of the early-stage LUAD patients from TCGA. [file 12920_2022_1413_MOESM3_ESM.docx]

**Additional file 3: Table S3** The clustering statuses of the early-stage LUAD patients from TCGA.

| Clustering | Patient ID |
| --- | --- |
| C1 | TCGA-MP-A4TF, TCGA-49-6742, TCGA-55-7284, TCGA-4B-A93V, TCGA-62-A46P, TCGA-55-7907, TCGA-55-8513, TCGA-L9-A50W, TCGA-44-A47A, TCGA-50-5931, TCGA-55-A4DF, TCGA-J2-8194, TCGA-50-5066, TCGA-55-7574, TCGA-55-7913, TCGA-49-4510, TCGA-55-8208, TCGA-55-8096, TCGA-50-5068, TCGA-78-7539, TCGA-MP-A4TA, TCGA-86-8669, TCGA-64-5778, TCGA-49-4486, TCGA-73-A9RS, TCGA-55-8092, TCGA-78-7161, TCGA-MP-A4TE, TCGA-44-7660, TCGA-44-A4SU, TCGA-62-A472, TCGA-86-8674, TCGA-62-8395, TCGA-55-7815, TCGA-55-7995, TCGA-44-3918, TCGA-62-A46S, TCGA-62-A470, TCGA-44-6146, TCGA-49-4506, TCGA-78-8648, TCGA-86-7955, TCGA-53-7626, TCGA-44-7671, TCGA-44-3919, TCGA-62-A46O, TCGA-50-5932, TCGA-44-2659, TCGA-50-5942, TCGA-78-7633, TCGA-75-7025, TCGA-50-5049, TCGA-86-A4D0, TCGA-69-8255, TCGA-38-A44F, TCGA-78-7150, TCGA-67-6216, TCGA-05-5423, TCGA-97-8174, TCGA-67-6215, TCGA-86-6851, TCGA-78-7148, TCGA-69-7761, TCGA-93-8067, TCGA-L9-A443, TCGA-78-7155, TCGA-69-7973, TCGA-44-6779, TCGA-44-2662, TCGA-64-5774, TCGA-78-7166, TCGA-49-AAR9, TCGA-44-8119, TCGA-44-A47B, TCGA-L9-A444, TCGA-91-6831, TCGA-91-6828, TCGA-MP-A4TJ, TCGA-44-A47G, TCGA-86-8585, TCGA-05-4422, TCGA-91-6840, TCGA-95-7944, TCGA-67-3774, TCGA-44-8117, TCGA-44-7669, TCGA-69-7764, TCGA-44-A4SS, TCGA-86-A4P7, TCGA-55-8619, TCGA-91-6836, TCGA-55-8507, TCGA-86-8668, TCGA-69-8253, TCGA-67-3773, TCGA-44-2668, TCGA-91-8497, TCGA-55-6543, TCGA-97-8179, TCGA-L4-A4E6, TCGA-55-7725, TCGA-55-1592, TCGA-05-4426, TCGA-50-5939, TCGA-55-8087, TCGA-95-7948, TCGA-95-7947, TCGA-55-A494, TCGA-97-7941, TCGA-05-5425, TCGA-55-7573, TCGA-91-7771, TCGA-91-8496, TCGA-S2-AA1A, TCGA-55-8621, TCGA-55-8514, TCGA-38-4630, TCGA-93-7348, TCGA-97-8172, TCGA-55-A57B, TCGA-55-8203, TCGA-97-7937, TCGA-L9-A7SV, TCGA-55-7903, TCGA-97-A4M6, TCGA-95-7567, TCGA-67-3772, TCGA-L4-A4E5, TCGA-05-4403, TCGA-69-A59K, TCGA-44-6145, TCGA-55-A492, TCGA-55-8091, TCGA-97-A4M1, TCGA-NJ-A55R, TCGA-91-A4BD, TCGA-55-A4DG, TCGA-67-3771, TCGA-67-3770, TCGA-97-A4LX, TCGA-55-8508, TCGA-44-4112, TCGA-97-A4M2, TCGA-97-8552, TCGA-55-A491, TCGA-55-A48Y, TCGA-97-A4M5, TCGA-97-A4M0, TCGA-50-5935, TCGA-86-8358, TCGA-99-AA5R, TCGA-55-7576, TCGA-05-5428, TCGA-69-7763, TCGA-44-7659, TCGA-44-6148, TCGA-55-7728, TCGA-55-7724, TCGA-44-7672, TCGA-55-6984, TCGA-05-4433, TCGA-05-4397, TCGA-86-8073, TCGA-MP-A4TH, TCGA-05-4432, TCGA-97-7547, TCGA-55-7570, TCGA-50-8460, TCGA-44-6147, TCGA-55-8206, TCGA-86-A456, TCGA-55-8085, TCGA-05-4420, TCGA-J2-A4AG, TCGA-86-8076, TCGA-55-7910, TCGA-53-A4EZ, TCGA-J2-A4AE, TCGA-44-7667, TCGA-99-8028, TCGA-50-8457, TCGA-49-AARN, TCGA-86-8054, TCGA-86-7713, TCGA-44-2661, TCGA-44-3398, TCGA-44-3917, TCGA-73-7498, TCGA-78-7540, TCGA-62-A471, TCGA-62-8397, TCGA-75-5147, TCGA-44-2657, TCGA-05-4389, TCGA-55-6971, TCGA-NJ-A4YQ, TCGA-05-4249, TCGA-78-7537, TCGA-55-6972, TCGA-49-4514, TCGA-62-A46R, TCGA-64-1676, TCGA-MP-A4SW, TCGA-44-6778, TCGA-78-7159, TCGA-55-1596, TCGA-55-6987, TCGA-NJ-A4YF, TCGA-62-A46V, TCGA-78-7162, TCGA-49-AAR2, TCGA-MP-A5C7, TCGA-55-6642, TCGA-75-6206, TCGA-44-6776, TCGA-MP-A4T4, TCGA-MP-A4SV, TCGA-55-6983, TCGA-80-5608, TCGA-55-6986, TCGA-78-7153, TCGA-49-AAR0, TCGA-49-AARQ, TCGA-78-8640. |
| C2 | TCGA-64-5781, TCGA-05-4424, TCGA-55-6982, TCGA-55-6979, TCGA-50-5946, TCGA-78-8660, TCGA-55-8299, TCGA-05-4382, TCGA-55-7281, TCGA-MP-A4TK, TCGA-49-4505, TCGA-86-A4JF, TCGA-55-8205, TCGA-49-AAQV, TCGA-55-8090, TCGA-91-6847, TCGA-78-7152, TCGA-73-7499, TCGA-71-6725, TCGA-71-8520, TCGA-86-8075, TCGA-95-8039, TCGA-55-8301, TCGA-97-8175, TCGA-44-7661, TCGA-69-8453, TCGA-49-AARE, TCGA-50-8459, TCGA-44-6775, TCGA-50-5055, TCGA-O1-A52J, TCGA-49-AARO, TCGA-95-7039, TCGA-49-AAR3, TCGA-75-7027, TCGA-78-8662, TCGA-55-A490, TCGA-86-8055, TCGA-69-7978, TCGA-35-4123, TCGA-69-7760, TCGA-44-7662, TCGA-35-4122, TCGA-73-4668, TCGA-44-8120, TCGA-86-6562, TCGA-67-6217, TCGA-38-7271, TCGA-78-7542, TCGA-38-4629, TCGA-05-4390, TCGA-69-7979, TCGA-69-7980, TCGA-64-1681, TCGA-05-4417, TCGA-55-8511, TCGA-55-8097, TCGA-L9-A8F4, TCGA-55-8302, TCGA-97-8177, TCGA-55-8204, TCGA-93-A4JQ, TCGA-55-8614, TCGA-55-8510, TCGA-49-4501, TCGA-95-A4VN, TCGA-78-7147, TCGA-55-7994, TCGA-86-7954, TCGA-05-4405, TCGA-97-A4M7, TCGA-49-4488, TCGA-55-7726, TCGA-L9-A743, TCGA-49-6767, TCGA-93-7347, TCGA-49-4487, TCGA-86-8280, TCGA-55-8089, TCGA-05-4430, TCGA-05-4427, TCGA-78-7535, TCGA-83-5908, TCGA-MN-A4N1, TCGA-86-8671, TCGA-64-5815, TCGA-86-8279, TCGA-55-8207, TCGA-44-6777, TCGA-86-7953, TCGA-38-4628, TCGA-38-4627, TCGA-MN-A4N4, TCGA-55-6985, TCGA-91-6829, TCGA-50-6597, TCGA-50-6590, TCGA-44-2665, TCGA-78-7143, TCGA-73-4658, TCGA-49-6744, TCGA-50-5944, TCGA-75-5125, TCGA-97-7553, TCGA-55-6980, TCGA-NJ-A4YG, TCGA-78-8655, TCGA-80-5611, TCGA-38-4625, TCGA-78-7163. |

*LUAD, lung adenocarcinoma; TCGA, the Cancer Genome Atlas.*
