# Supplementary figures and images for: Construction and experimental validation of an acetylation-related gene signature to evaluate the recurrence and immunotherapeutic response in early-stage lung adenocarcinoma
Source: BMC Med Genomics. 2022 Dec 11;15:254. doi: 10.1186/s12920-022-01413-7 (PMC9741798; doi:10.1186/s12920-022-01413-7)

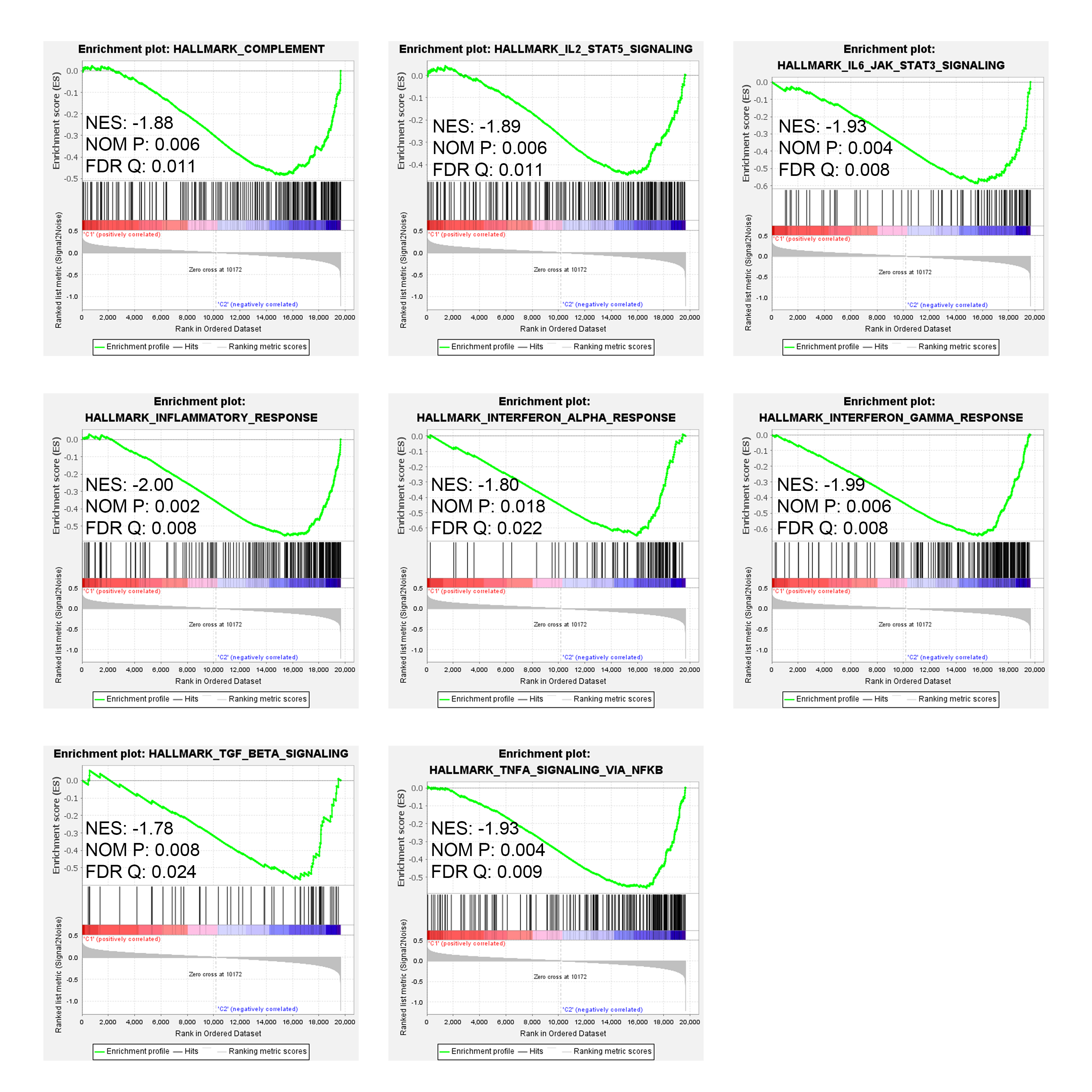

Supplement: Supplementary file 4 — Additional file 4. Fig. S1: The GSEA indicated that the early-stage LUAD patients in C2 subgroup exhibited stronger immune response. [file 12920_2022_1413_MOESM4_ESM.tif]

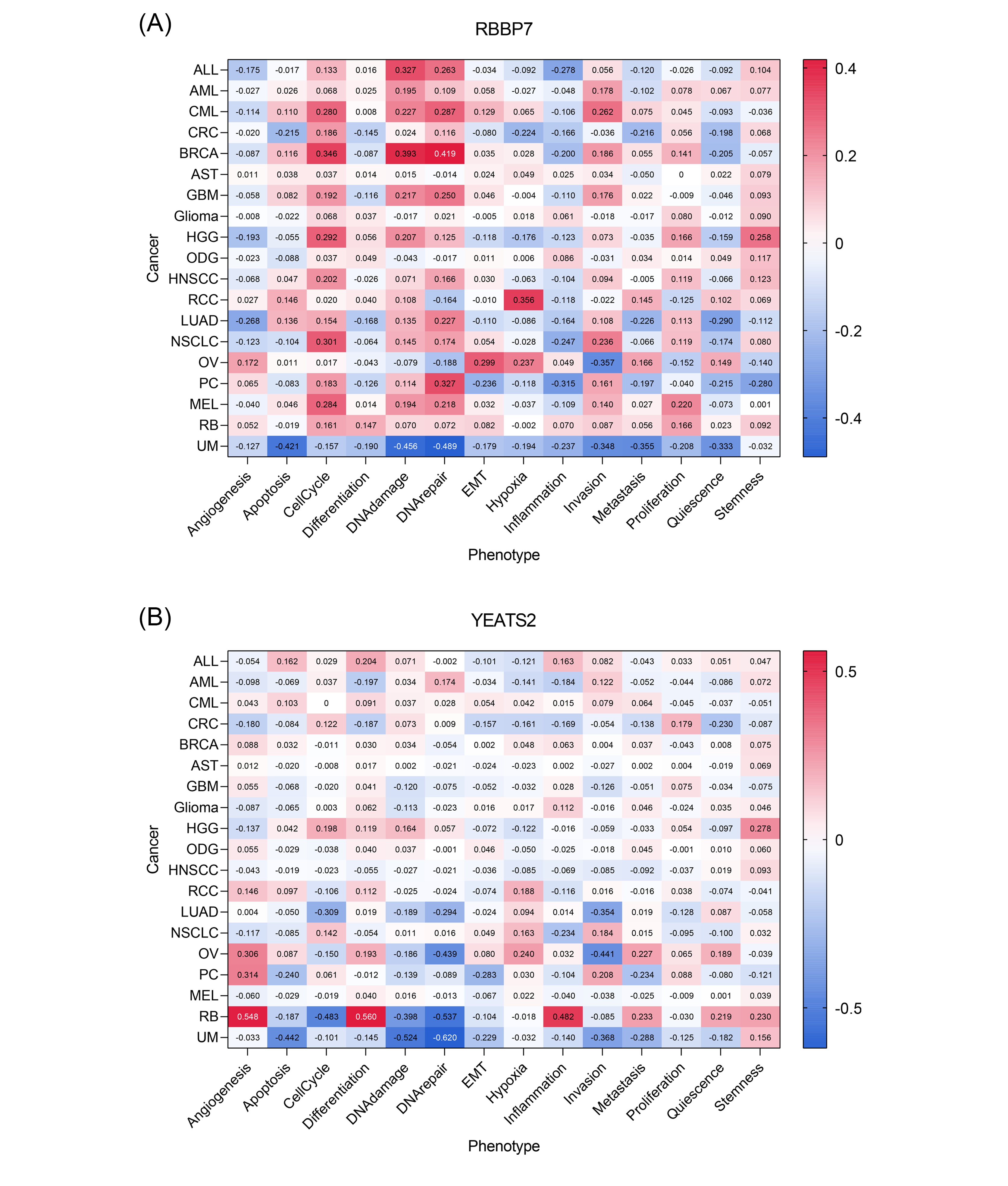

Supplement: Supplementary file 8 — Additional file 8. Fig. S2: The pan-cancer analyses of RBBP7 (A) and YEATS2 (B) from the single-cell level. [file 12920_2022_1413_MOESM8_ESM.tif]

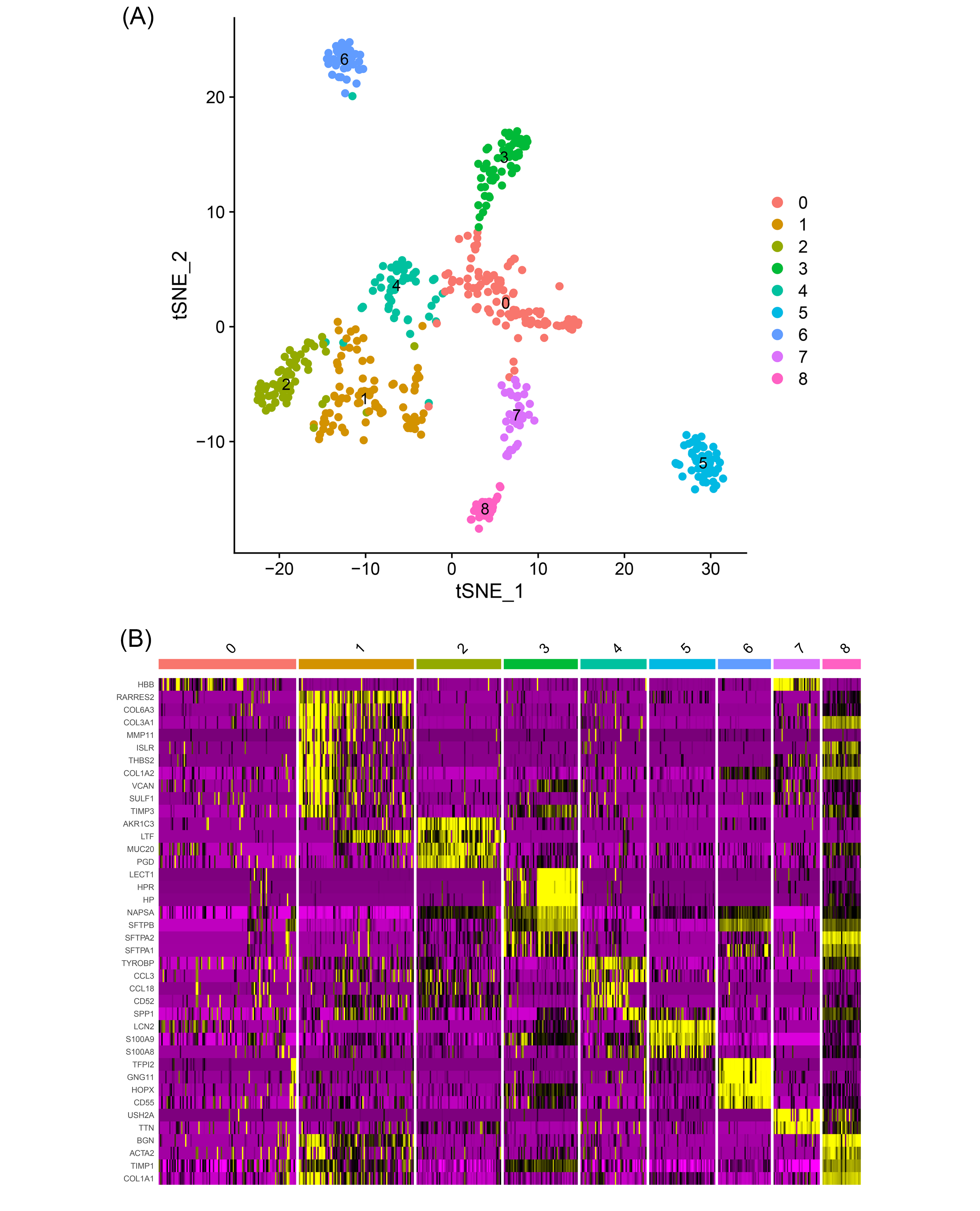

Supplement: Supplementary file 10 — Additional file 10. Fig. S3: The process of single-cell RNA sequencing analyses. A 520 cell samples from tumor tissue were divided into 9 cell clusters. B The expression level of the marker genes in each cluster. The colors ranging from purple to yellow represented the expression values from low to high. [file 12920_2022_1413_MOESM10_ESM.tif]
