# Supplementary material for: Construction and experimental validation of an acetylation-related gene signature to evaluate the recurrence and immunotherapeutic response in early-stage lung adenocarcinoma
Source: BMC Med Genomics. 2022 Dec 11;15:254. doi: 10.1186/s12920-022-01413-7 (PMC9741798; doi:10.1186/s12920-022-01413-7)
Supplement: Supplementary file 5 — Additional file 5. Table S4: The comparison of the immune cell infiltration proportion between C1 and C2 subgroups. [file 12920_2022_1413_MOESM5_ESM.docx]

**Additional file 5 Table S4** The comparison of the immune cell infiltration proportion between C1 and C2 subgroups.

| Immune cell | Algorithm | Mean infiltration level in C1 | Mean infiltration level in C2 | Fold change (C2/C1) | P value |
| --- | --- | --- | --- | --- | --- |
| B cell | TIMER | 0.090389245 | 0.081606305 | 0.902832024 | >0.05 |
| T cell CD4+ | TIMER | 0.132010864 | 0.158460035 | 1.200356018 | **<0.01** |
| T cell CD8+ | TIMER | 0.153310267 | 0.190557367 | 1.242952418 | **<0.01** |
| Neutrophil | TIMER | 0.10880316 | 0.152935033 | 1.405612052 | **<0.001** |
| Macrophage | TIMER | 0.06015034 | 0.089715433 | 1.491519965 | **<0.001** |
| Myeloid dendritic cell | TIMER | 0.422473521 | 0.575825721 | 1.362986536 | **<0.001** |
| B cell naive | CIBERSORT | 0.014305995 | 0.008951346 | 0.625705937 | **<0.01** |
| B cell memory | CIBERSORT | 0.019792826 | 0.019204129 | 0.970257052 | >0.05 |
| B cell plasma | CIBERSORT | 0.086062716 | 0.045661365 | 0.530559191 | **<0.001** |
| T cell CD8+ | CIBERSORT | 0.095705121 | 0.089681523 | 0.93706086 | >0.05 |
| T cell CD4+ naive | CIBERSORT | 0.000142358 | 0 | 0 | >0.05 |
| T cell CD4+ memory resting | CIBERSORT | 0.128927056 | 0.12156816 | 0.942922019 | >0.05 |
| T cell CD4+ memory activated | CIBERSORT | 0.005369893 | 0.009053066 | 1.685893183 | **<0.05** |
| T cell follicular helper | CIBERSORT | 0.056535514 | 0.045386834 | 0.802802182 | **<0.01** |
| T cell regulatory (Tregs) | CIBERSORT | 0.020363047 | 0.022002135 | 1.080493258 | >0.05 |
| T cell gamma delta | CIBERSORT | 0.003819808 | 0.003376524 | 0.883951235 | >0.05 |
| NK cell resting | CIBERSORT | 0.005730958 | 0.007020593 | 1.225029567 | >0.05 |
| NK cell activated | CIBERSORT | 0.039176563 | 0.034289144 | 0.875246356 | **<0.05** |
| Monocyte | CIBERSORT | 0.0323234 | 0.039221138 | 1.213397662 | >0.05 |
| Macrophage M0 | CIBERSORT | 0.077829032 | 0.102562886 | 1.317797271 | **<0.01** |
| Macrophage M1 | CIBERSORT | 0.055166152 | 0.061359834 | 1.112273229 | >0.05 |
| Macrophage M2 | CIBERSORT | 0.260621112 | 0.288759188 | 1.107965451 | **<0.01** |
| Myeloid dendritic cell resting | CIBERSORT | 0.018865136 | 0.025663179 | 1.360349536 | **<0.05** |
| Myeloid dendritic cell activated | CIBERSORT | 0.017238583 | 0.017940738 | 1.040731596 | >0.05 |
| Mast cell activated | CIBERSORT | 0.041024008 | 0.036619701 | 0.892640743 | >0.05 |
| Mast cell resting | CIBERSORT | 0.012644466 | 0.014015702 | 1.108445544 | >0.05 |
| Eosinophil | CIBERSORT | 0.000146977 | 2.34E-05 | 0.159208584 | >0.05 |
| Neutrophil | CIBERSORT | 0.008209279 | 0.007639402 | 0.930581358 | >0.05 |
