# Supplementary material for: Construction and experimental validation of an acetylation-related gene signature to evaluate the recurrence and immunotherapeutic response in early-stage lung adenocarcinoma
Source: BMC Med Genomics. 2022 Dec 11;15:254. doi: 10.1186/s12920-022-01413-7 (PMC9741798; doi:10.1186/s12920-022-01413-7)
Supplement: Supplementary file 6 — Additional file 6. Table S5: The importance of genes in the PPI network. [file 12920_2022_1413_MOESM6_ESM.docx]

**Additional file 6: Table S5** The importance of genes in the PPI network.

| Genes | MCC | DMNC | MNC | Degree | EPC | BottleNeck | EcCentricity | Closeness | Radiality | Betweenness | Stress | ClusteringCoefficient |
| --- | --- | --- | --- | --- | --- | --- | --- | --- | --- | --- | --- | --- |
| KAT2B | 9.22E+13 | 0.52432 | 76 | 77 | 36.501 | 13 | 0.33333 | 110.33333 | 4.54483 | 2658.34685 | 24346 | 0.2823 |
| TP53 | 1.82E+07 | 0.36503 | 73 | 74 | 33.846 | 36 | 0.33333 | 108 | 4.48966 | 3294.00477 | 27190 | 0.19882 |
| KAT2A | 9.22E+13 | 0.55733 | 69 | 69 | 35.626 | 2 | 0.33333 | 106.16667 | 4.48276 | 1918.33565 | 20116 | 0.31756 |
| HDAC1 | 2.12E+11 | 0.47135 | 58 | 58 | 32.051 | 5 | 0.33333 | 100.16667 | 4.38621 | 1167.65101 | 12092 | 0.28373 |
| WDR5 | 9.22E+13 | 0.68114 | 57 | 58 | 34.687 | 7 | 0.33333 | 100.16667 | 4.38621 | 921.79879 | 8632 | 0.39806 |
| TRRAP | 9.22E+13 | 0.68425 | 57 | 57 | 35.31 | 8 | 0.33333 | 99.16667 | 4.35862 | 691.7494 | 7376 | 0.41416 |
| KAT5 | 9.22E+13 | 0.57839 | 52 | 52 | 32.677 | 2 | 0.33333 | 96.83333 | 4.33103 | 813.60332 | 8448 | 0.36048 |
| HDAC2 | 1.06E+11 | 0.57091 | 45 | 45 | 29.89 | 6 | 0.33333 | 93.5 | 4.28966 | 546.22741 | 7706 | 0.37273 |
| RUVBL2 | 9.22E+13 | 0.80201 | 42 | 42 | 32.254 | 5 | 0.33333 | 91.5 | 4.24828 | 346.1362 | 4826 | 0.53542 |
| RUVBL1 | 9.22E+13 | 0.80548 | 42 | 42 | 31.622 | 4 | 0.33333 | 90.66667 | 4.21379 | 341.70791 | 4264 | 0.53775 |
| YEATS2 | 9.22E+13 | 0.81244 | 42 | 42 | 31.169 | 1 | 0.33333 | 89.33333 | 4.15862 | 191.55634 | 2238 | 0.54239 |
| MORF4L1 | 9.22E+13 | 0.83167 | 40 | 40 | 30.161 | 1 | 0.33333 | 89.83333 | 4.2069 | 225.69997 | 2758 | 0.5641 |
| ACTL6A | 9.22E+13 | 0.89193 | 39 | 39 | 30.656 | 1 | 0.33333 | 89.66667 | 4.21379 | 178.59427 | 2502 | 0.60999 |
| DMAP1 | 9.22E+13 | 0.95585 | 35 | 37 | 27.909 | 4 | 0.33333 | 87.5 | 4.15172 | 692.35274 | 5248 | 0.60511 |
| MAP3K7 | 9.22E+13 | 0.68546 | 35 | 37 | 28.279 | 5 | 0.33333 | 89.5 | 4.23448 | 1051.74777 | 8858 | 0.43544 |
| ING3 | 9.22E+13 | 0.88176 | 36 | 36 | 28.841 | 1 | 0.33333 | 88.16667 | 4.1931 | 209.65224 | 3468 | 0.61905 |
| EP400 | 9.22E+13 | 0.96296 | 35 | 35 | 30.411 | 1 | 0.33333 | 87.66667 | 4.18621 | 72.87582 | 1598 | 0.68235 |
| MCRS1 | 9.22E+13 | 0.85463 | 34 | 34 | 28.604 | 1 | 0.33333 | 85.83333 | 4.12414 | 176.07945 | 2134 | 0.61141 |
| CHD4 | 6.92E+09 | 0.67025 | 34 | 34 | 27.881 | 1 | 0.33333 | 87.33333 | 4.18621 | 285.6789 | 3706 | 0.4795 |
| RBBP4 | 1.80E+08 | 0.62388 | 33 | 33 | 26.173 | 1 | 0.33333 | 86.66667 | 4.17241 | 186.83204 | 3398 | 0.45076 |
| KAT6B | 9.22E+13 | 0.68155 | 33 | 33 | 26.719 | 2 | 0.33333 | 87 | 4.18621 | 331.80296 | 3908 | 0.49242 |
| FOXO1 | 36714 | 0.41984 | 32 | 32 | 20.602 | 1 | 0.33333 | 85.5 | 4.13793 | 387.52879 | 4006 | 0.30645 |
| RBBP7 | 9.63E+07 | 0.59938 | 32 | 32 | 25.2 | 5 | 0.33333 | 85.83333 | 4.15172 | 188.58758 | 3430 | 0.4375 |
| BRCA1 | 883722 | 0.50143 | 31 | 31 | 23.483 | 1 | 0.33333 | 85.16667 | 4.13793 | 280.24245 | 3908 | 0.36989 |
| MORF4L2 | 9.22E+13 | 1.05111 | 30 | 31 | 28.221 | 2 | 0.33333 | 84.5 | 4.11034 | 315.50455 | 2506 | 0.73333 |
| POLE3 | 9.22E+13 | 0.86585 | 31 | 31 | 26.146 | 3 | 0.33333 | 85 | 4.13103 | 138.1203 | 1804 | 0.63871 |
| MRGBP | 9.22E+13 | 1.02619 | 31 | 31 | 27.619 | 1 | 0.33333 | 83.5 | 4.06897 | 28.20911 | 546 | 0.75699 |
| KANSL1 | 9.22E+13 | 0.91541 | 31 | 31 | 27.533 | 1 | 0.33333 | 84 | 4.08966 | 68.23834 | 1170 | 0.67527 |
| FOXO3 | 50670 | 0.49011 | 30 | 30 | 18.468 | 5 | 0.33333 | 84.33333 | 4.11724 | 261.19157 | 3010 | 0.36552 |
| MLLT3 | 1.45E+10 | 0.7367 | 30 | 30 | 25.296 | 2 | 0.33333 | 84.5 | 4.12414 | 135.652 | 1890 | 0.54943 |
| HCFC1 | 6.31E+09 | 0.67265 | 29 | 29 | 26.198 | 4 | 0.25 | 83.25 | 4.08276 | 169.1992 | 2364 | 0.50739 |
| SMAD4 | 470106 | 0.5199 | 28 | 28 | 21.151 | 2 | 0.33333 | 83.5 | 4.11034 | 216.73138 | 3258 | 0.39683 |
| MAPK3 | 19126 | 0.42285 | 28 | 28 | 16.498 | 2 | 0.25 | 80.58333 | 3.97241 | 517.93245 | 5108 | 0.32275 |
| BRD8 | 9.22E+13 | 1.01554 | 28 | 28 | 26.967 | 1 | 0.33333 | 84 | 4.13103 | 109.01483 | 1588 | 0.77513 |
| TAF6 | 4.45E+13 | 0.91071 | 27 | 27 | 25.592 | 1 | 0.33333 | 82.33333 | 4.07586 | 57.73689 | 1240 | 0.7037 |
| HDAC5 | 5119371 | 0.6015 | 26 | 27 | 22.702 | 2 | 0.33333 | 83.66667 | 4.13103 | 355.97108 | 3586 | 0.4359 |
| SMARCB1 | 6705876 | 0.61574 | 27 | 27 | 23.597 | 1 | 0.33333 | 83.16667 | 4.11034 | 186.78934 | 2368 | 0.47578 |
| SRCAP | 9.22E+13 | 0.94758 | 27 | 27 | 25.679 | 2 | 0.25 | 80.58333 | 3.9931 | 82.17289 | 1040 | 0.73219 |
| TAF4 | 4.45E+13 | 0.92781 | 26 | 26 | 24.176 | 1 | 0.33333 | 81.83333 | 4.06897 | 52.91837 | 1180 | 0.72615 |
| MLLT1 | 7.23E+09 | 0.71158 | 26 | 26 | 24.985 | 1 | 0.25 | 79 | 3.93793 | 94.11611 | 1398 | 0.55692 |
| TAF12 | 4.46E+13 | 0.89932 | 25 | 25 | 22.04 | 1 | 0.33333 | 81.66667 | 4.07586 | 69.77886 | 1232 | 0.71333 |
| IL1B | 15976 | 0.48424 | 23 | 25 | 13.808 | 2 | 0.25 | 77.58333 | 3.88966 | 472.45644 | 3736 | 0.33333 |
| ATF2 | 903918 | 0.48748 | 25 | 25 | 20.076 | 2 | 0.33333 | 82.66667 | 4.11724 | 338.63308 | 3554 | 0.38667 |
| MSL1 | 9.22E+13 | 1.17115 | 24 | 25 | 24.611 | 1 | 0.33333 | 80.33333 | 4.02069 | 124.35473 | 788 | 0.86667 |
| KANSL2 | 9.22E+13 | 1.11365 | 25 | 25 | 24.82 | 1 | 0.33333 | 80.83333 | 4.04138 | 14.58259 | 236 | 0.88333 |
| KANSL3 | 9.22E+13 | 1.11365 | 25 | 25 | 25.126 | 1 | 0.33333 | 80.83333 | 4.04138 | 14.58259 | 236 | 0.88333 |
| KAT7 | 3.74E+10 | 0.76064 | 25 | 25 | 24.86 | 2 | 0.33333 | 82.66667 | 4.11724 | 125.58773 | 2074 | 0.60333 |
| TADA2A | 4.46E+13 | 0.92791 | 24 | 24 | 22.364 | 1 | 0.25 | 75.83333 | 3.83448 | 24.15088 | 250 | 0.74638 |
| TAF9 | 2.22E+13 | 0.86195 | 23 | 23 | 21.414 | 1 | 0.25 | 75.66667 | 3.84138 | 61.31623 | 588 | 0.70356 |
| TAF10 | 4.46E+13 | 0.96848 | 23 | 23 | 21.323 | 1 | 0.33333 | 80.16667 | 4.04138 | 48.45216 | 976 | 0.79051 |
| ZZZ3 | 1.74E+11 | 0.83774 | 23 | 23 | 23.149 | 1 | 0.33333 | 79 | 3.9931 | 53.52991 | 788 | 0.68379 |
| ENY2 | 4.45E+13 | 0.97785 | 21 | 22 | 20.535 | 3 | 0.25 | 74.83333 | 3.82069 | 294.02597 | 3750 | 0.74892 |
| TAF5L | 4.46E+13 | 0.99228 | 22 | 22 | 22.182 | 1 | 0.25 | 76.08333 | 3.87586 | 20.05517 | 354 | 0.82251 |
| TRAF6 | 14023 | 0.49175 | 21 | 22 | 12.919 | 4 | 0.25 | 75.75 | 3.85517 | 384.76243 | 3178 | 0.37662 |
| FOXP3 | 15462 | 0.53132 | 21 | 21 | 17.484 | 6 | 0.33333 | 79.83333 | 4.05517 | 219.49926 | 2548 | 0.44762 |
| TAF5 | 4.19E+13 | 0.96655 | 21 | 21 | 19.77 | 1 | 0.33333 | 78.83333 | 4.01379 | 41.12007 | 878 | 0.81429 |
| TAF6L | 4.46E+13 | 1.06264 | 21 | 21 | 20.692 | 1 | 0.25 | 74.33333 | 3.81379 | 4.78987 | 112 | 0.89524 |
| NFKB1 | 20804 | 0.522 | 20 | 20 | 15.399 | 1 | 0.25 | 77.08333 | 3.95172 | 86.7872 | 1022 | 0.44737 |
| TAF2 | 4.19E+13 | 1.02557 | 20 | 20 | 18.152 | 1 | 0.33333 | 78.33333 | 4.0069 | 31.85803 | 810 | 0.87895 |
| ATXN7L3 | 4.45E+13 | 1.00715 | 20 | 20 | 18.84 | 1 | 0.25 | 73.5 | 3.7931 | 136.22635 | 2590 | 0.86316 |
| FOXO4 | 6877 | 0.44224 | 19 | 20 | 13.301 | 1 | 0.25 | 76.58333 | 3.91724 | 153.68595 | 1236 | 0.34737 |
| TADA1 | 4.45E+13 | 1.09221 | 19 | 19 | 19.237 | 1 | 0.25 | 73 | 3.78621 | 1.36343 | 26 | 0.95322 |
| SUPT7L | 4.45E+13 | 1.09221 | 19 | 19 | 18.857 | 1 | 0.25 | 73 | 3.78621 | 1.36343 | 26 | 0.95322 |
| LEF1 | 698 | 0.39534 | 19 | 19 | 15.173 | 1 | 0.33333 | 78.16667 | 4.01379 | 102.83684 | 1136 | 0.34503 |
| IKBKB | 12630 | 0.49951 | 18 | 18 | 13.686 | 1 | 0.25 | 74.08333 | 3.84138 | 54.79815 | 550 | 0.44444 |
| NCOA3 | 12199 | 0.5564 | 16 | 17 | 17.86 | 2 | 0.33333 | 77.5 | 4.01379 | 137.47418 | 1586 | 0.45588 |
| NFKBIA | 22008 | 0.62334 | 17 | 17 | 13.829 | 1 | 0.25 | 74.58333 | 3.87586 | 77.60713 | 986 | 0.56618 |
| AKT2 | 7872 | 0.50191 | 17 | 17 | 12.317 | 1 | 0.25 | 72.66667 | 3.78621 | 68.23479 | 672 | 0.45588 |
| VPS72 | 8.77E+10 | 0.97954 | 17 | 17 | 19.899 | 1 | 0.25 | 71.16667 | 3.73793 | 2.69672 | 40 | 0.88971 |
| CTBP1 | 172329 | 0.65512 | 16 | 17 | 16.03 | 1 | 0.33333 | 77.5 | 4.01379 | 82.33466 | 922 | 0.53676 |
| SIRT3 | 20365 | 0.56083 | 15 | 16 | 14.72 | 2 | 0.33333 | 77.5 | 4.02759 | 281.25058 | 2770 | 0.46667 |
| OGT | 5116 | 0.43429 | 13 | 15 | 13.01 | 1 | 0.33333 | 74.33333 | 3.91034 | 210.634 | 1970 | 0.32381 |
| AKT3 | 7387 | 0.54053 | 14 | 15 | 10.695 | 1 | 0.25 | 71.33333 | 3.75862 | 93.62217 | 858 | 0.45714 |
| SPI1 | 606 | 0.48423 | 14 | 14 | 12.347 | 1 | 0.33333 | 75.5 | 3.97241 | 49.53616 | 728 | 0.47253 |
| MECP2 | 46260 | 0.58558 | 14 | 14 | 14.699 | 1 | 0.25 | 73.58333 | 3.87586 | 18.53508 | 272 | 0.57143 |
| MBTD1 | 4.79E+08 | 0.85585 | 14 | 14 | 16.738 | 1 | 0.25 | 69.83333 | 3.72414 | 3.50432 | 74 | 0.83516 |
| BRCA2 | 7608 | 0.57432 | 14 | 14 | 15.26 | 1 | 0.33333 | 75.83333 | 3.98621 | 34.54336 | 564 | 0.56044 |
| MTA2 | 126246 | 0.65315 | 14 | 14 | 13.211 | 1 | 0.25 | 72.08333 | 3.81379 | 20.54616 | 238 | 0.63736 |
| BRPF3 | 6250 | 0.50675 | 14 | 14 | 14.3 | 1 | 0.25 | 73.58333 | 3.87586 | 36.69677 | 728 | 0.49451 |
| TWIST1 | 11328 | 0.58558 | 14 | 14 | 13.746 | 1 | 0.33333 | 75.16667 | 3.95862 | 32.99733 | 616 | 0.57143 |
| NAA50 | 5107 | 0.48295 | 12 | 13 | 9.056 | 5 | 0.25 | 69 | 3.70345 | 366.12206 | 6484 | 0.42308 |
| NFYA | 6024 | 0.56202 | 13 | 13 | 13.254 | 1 | 0.33333 | 74.33333 | 3.93793 | 18.01589 | 206 | 0.5641 |
| NAA10 | 5208 | 0.52686 | 12 | 12 | 9.345 | 1 | 0.25 | 69.66667 | 3.74483 | 452.15476 | 7310 | 0.54545 |
| TXN | 558 | 0.52686 | 12 | 12 | 8.574 | 1 | 0.25 | 68.33333 | 3.67586 | 23.85229 | 208 | 0.54545 |
| NFYB | 6576 | 0.60003 | 12 | 12 | 13.02 | 1 | 0.33333 | 73.83333 | 3.93103 | 18.13764 | 300 | 0.62121 |
| CHEK1 | 3842 | 0.5854 | 12 | 12 | 14.758 | 1 | 0.33333 | 74.66667 | 3.96552 | 44.9719 | 596 | 0.60606 |
| BAZ1A | 1476 | 0.55613 | 12 | 12 | 15.273 | 1 | 0.25 | 70.58333 | 3.78621 | 8.45711 | 144 | 0.57576 |
| GATA2 | 217 | 0.51877 | 10 | 11 | 10.467 | 1 | 0.33333 | 73.16667 | 3.91724 | 30.3174 | 262 | 0.47273 |
| HAT1 | 1686 | 0.57691 | 11 | 11 | 14.105 | 1 | 0.25 | 72.58333 | 3.88966 | 30.49479 | 688 | 0.61818 |
| NFYC | 6534 | 0.62782 | 11 | 11 | 11.77 | 1 | 0.33333 | 73.16667 | 3.91724 | 12.09104 | 234 | 0.67273 |
| TRADD | 10332 | 0.64479 | 11 | 11 | 8.082 | 1 | 0.25 | 69.25 | 3.73793 | 21.71176 | 276 | 0.69091 |
| FADD | 11664 | 0.67872 | 11 | 11 | 9.127 | 1 | 0.25 | 69.58333 | 3.75172 | 21.77257 | 304 | 0.72727 |
| PHF20L1 | 1202 | 0.55995 | 11 | 11 | 13.85 | 2 | 0.25 | 69.25 | 3.73103 | 24.23304 | 362 | 0.6 |
| PAXIP1 | 840 | 0.61085 | 11 | 11 | 12.08 | 1 | 0.33333 | 73.33333 | 3.92414 | 5.96368 | 84 | 0.65455 |
| IRF4 | 240 | 0.53872 | 10 | 10 | 10.389 | 1 | 0.33333 | 73.5 | 3.94483 | 21.48471 | 392 | 0.6 |
| NAA15 | 5088 | 0.64439 | 9 | 9 | 5.971 | 1 | 0.25 | 66.83333 | 3.66897 | 231.00376 | 4260 | 0.75 |
| PRKAA1 | 131 | 0.4082 | 8 | 9 | 8.261 | 2 | 0.25 | 68.58333 | 3.73793 | 296.46192 | 2084 | 0.38889 |
| NCOA1 | 390 | 0.57279 | 9 | 9 | 11.852 | 1 | 0.33333 | 72.83333 | 3.93103 | 4.8658 | 106 | 0.66667 |
| TXNIP | 150 | 0.5012 | 9 | 9 | 8.052 | 1 | 0.25 | 68.58333 | 3.73793 | 13.28327 | 200 | 0.58333 |
| NOS1 | 38 | 0.40246 | 7 | 9 | 6.813 | 2 | 0.25 | 65.25 | 3.6 | 38.34762 | 314 | 0.30556 |
| CRTC2 | 41 | 0.37904 | 8 | 9 | 7.362 | 1 | 0.33333 | 70.16667 | 3.82069 | 41.29151 | 546 | 0.36111 |
| NAA20 | 5064 | 0.69977 | 8 | 8 | 4.348 | 1 | 0.25 | 64.25 | 3.57241 | 95.9429 | 1856 | 0.85714 |
| PARK7 | 84 | 0.46652 | 8 | 8 | 7.776 | 1 | 0.25 | 67.58333 | 3.71034 | 11.46291 | 142 | 0.57143 |
| MAP2K6 | 67 | 0.51223 | 7 | 8 | 7.737 | 1 | 0.25 | 68.5 | 3.75172 | 76.35359 | 512 | 0.5 |
| NAA30 | 5040 | 0.76834 | 7 | 7 | 2.461 | 1 | 0.2 | 50.13333 | 2.82759 | 0 | 0 | 1 |
| NAA40 | 5040 | 0.76834 | 7 | 7 | 2.568 | 1 | 0.2 | 50.13333 | 2.82759 | 0 | 0 | 1 |
| NAA35 | 5040 | 0.76834 | 7 | 7 | 2.609 | 1 | 0.2 | 50.13333 | 2.82759 | 0 | 0 | 1 |
| NAA25 | 5040 | 0.76834 | 7 | 7 | 2.365 | 1 | 0.2 | 50.13333 | 2.82759 | 0 | 0 | 1 |
| SET | 145 | 0.61814 | 6 | 7 | 9.836 | 1 | 0.25 | 68.75 | 3.78621 | 32.75018 | 816 | 0.61905 |
| TNFRSF1B | 146 | 0.51223 | 7 | 7 | 5.109 | 1 | 0.25 | 57.75 | 3.28276 | 3.47102 | 22 | 0.66667 |
| PRKAA2 | 128 | 0.47564 | 7 | 7 | 7.096 | 1 | 0.25 | 67.41667 | 3.71724 | 4.32288 | 66 | 0.61905 |
| PIN1 | 20 | 0.45378 | 5 | 7 | 7.309 | 2 | 0.25 | 68 | 3.74483 | 79.60948 | 596 | 0.33333 |
| ESCO1 | 246 | 0.5854 | 7 | 7 | 9.372 | 1 | 0.25 | 69.5 | 3.81379 | 12.32972 | 314 | 0.7619 |
| MUC1 | 44 | 0.43905 | 7 | 7 | 7.488 | 1 | 0.25 | 65.08333 | 3.6069 | 18.61794 | 236 | 0.57143 |
| SOX4 | 72 | 0.57059 | 6 | 6 | 7.284 | 1 | 0.25 | 65.58333 | 3.65517 | 0.82051 | 6 | 0.8 |
| PYGO2 | 36 | 0.47549 | 6 | 6 | 9.354 | 1 | 0.25 | 68.08333 | 3.76552 | 2.0586 | 24 | 0.66667 |
| PCGF2 | 122 | 0.52304 | 6 | 6 | 7.102 | 1 | 0.25 | 61.75 | 3.48276 | 0.97527 | 8 | 0.73333 |
| ARRB1 | 32 | 0.42794 | 6 | 6 | 5.428 | 2 | 0.25 | 63.91667 | 3.57241 | 39.03223 | 496 | 0.6 |
| SNCA | 18 | 0.45378 | 5 | 5 | 4.626 | 1 | 0.25 | 63.75 | 3.57931 | 2.19095 | 20 | 0.7 |
| IWS1 | 9 | 0.37893 | 4 | 5 | 6.668 | 1 | 0.25 | 62 | 3.50345 | 6.01276 | 102 | 0.4 |
| PIH1D1 | 7 | 0.46346 | 3 | 4 | 5.316 | 1 | 0.25 | 60.08333 | 3.44138 | 1.59906 | 24 | 0.5 |
| JADE3 | 8 | 0.37893 | 4 | 4 | 6.728 | 1 | 0.25 | 59.16667 | 3.4 | 0.15 | 4 | 0.66667 |
| JADE1 | 8 | 0.37893 | 4 | 4 | 5.934 | 1 | 0.25 | 59.16667 | 3.4 | 0.15 | 4 | 0.66667 |
| PHF14 | 6 | 0.2842 | 4 | 4 | 5.293 | 1 | 0.25 | 58 | 3.34483 | 1.13333 | 10 | 0.5 |
| NOC2L | 3 | 0.30779 | 2 | 3 | 5.194 | 1 | 0.25 | 66.25 | 3.73793 | 0.88642 | 36 | 0.33333 |
| SETD5 | 4 | 0.30898 | 3 | 3 | 5.222 | 1 | 0.25 | 57.41667 | 3.33793 | 0.10526 | 2 | 0.66667 |
| LIF | 6 | 0.46346 | 3 | 3 | 4.71 | 1 | 0.25 | 62.58333 | 3.55862 | 0 | 0 | 1 |
| MYOCD | 3 | 0 | 1 | 3 | 3.515 | 1 | 0.25 | 56.75 | 3.31034 | 1.22291 | 10 | 0 |
| KLF15 | 3 | 0.30779 | 2 | 3 | 4.852 | 1 | 0.25 | 65.25 | 3.69655 | 0.825 | 10 | 0.33333 |
| PIP4K2C | 3 | 0 | 1 | 3 | 3.166 | 1 | 0.25 | 54.33333 | 3.1931 | 10.63101 | 68 | 0 |
| RPS6KA4 | 3 | 0.30779 | 2 | 3 | 4.109 | 1 | 0.25 | 59.58333 | 3.44138 | 3.31311 | 30 | 0.33333 |
| CAMK1 | 3 | 0.30779 | 2 | 3 | 3.496 | 1 | 0.25 | 56.41667 | 3.30345 | 2.22097 | 20 | 0.33333 |
| APBB1 | 3 | 0.30779 | 2 | 3 | 4.869 | 1 | 0.25 | 64.83333 | 3.66207 | 3.04475 | 56 | 0.33333 |
| ARID5A | 6 | 0.46346 | 3 | 3 | 5.021 | 1 | 0.25 | 60 | 3.44828 | 0 | 0 | 1 |
| ZMPSTE24 | 2 | 0 | 1 | 2 | 2.913 | 1 | 0.25 | 52.66667 | 3.14483 | 0.63093 | 8 | 0 |
| MCM3AP | 2 | 0.30779 | 2 | 2 | 2.506 | 1 | 0.2 | 48.63333 | 2.83448 | 0 | 0 | 1 |
| ATAT1 | 2 | 0.30779 | 2 | 2 | 4.157 | 1 | 0.25 | 62.5 | 3.58621 | 0 | 0 | 1 |
| RAPGEF3 | 2 | 0.30779 | 2 | 2 | 2.029 | 1 | 0.2 | 51.16667 | 2.9931 | 0 | 0 | 1 |
| PER1 | 1 | 0 | 1 | 1 | 2.919 | 1 | 0.25 | 57.83333 | 3.3931 | 0 | 0 | 0 |
| NAP1L2 | 1 | 0 | 1 | 1 | 2.434 | 1 | 0.25 | 52.25 | 3.11724 | 0 | 0 | 0 |
| TRIM16 | 1 | 0 | 1 | 1 | 1.603 | 1 | 0.2 | 49.25 | 2.89655 | 0 | 0 | 0 |
| FLCN | 1 | 0 | 1 | 1 | 1.29 | 1 | 0.2 | 46.08333 | 2.74483 | 0 | 0 | 0 |
| EID1 | 1 | 0 | 1 | 1 | 2.714 | 1 | 0.25 | 61.33333 | 3.55172 | 0 | 0 | 0 |
| DIP2B | 1 | 0 | 1 | 1 | 2.218 | 1 | 0.25 | 53.25 | 3.15862 | 0 | 0 | 0 |
| DIP2A | 1 | 0 | 1 | 1 | 2.435 | 1 | 0.25 | 53.25 | 3.15862 | 0 | 0 | 0 |
| BAG6 | 1 | 0 | 1 | 1 | 1.383 | 1 | 0.2 | 48.58333 | 2.86207 | 0 | 0 | 0 |

*PPI, protein-protein interaction.*
