# Supplementary material for: Construction and experimental validation of an acetylation-related gene signature to evaluate the recurrence and immunotherapeutic response in early-stage lung adenocarcinoma
Source: BMC Med Genomics. 2022 Dec 11;15:254. doi: 10.1186/s12920-022-01413-7 (PMC9741798; doi:10.1186/s12920-022-01413-7)
Supplement: Supplementary file 7 — Additional file 7. Table S6: The univariate Cox regression of the Top 30 genes showing the highest degree. [file 12920_2022_1413_MOESM7_ESM.docx]

**Additional file 7: Table S6** The univariate Cox regression of the Top 30 genes showing the highest degree.

| id | HR (95%CI) | P-value |
| --- | --- | --- |
| YEATS2 | 1.017774685 (1.002774519~1.032999234) | 0.020034482 |
| RBBP7 | 1.008603594 (1.000979886~1.016285365) | 0.026900205 |
| ACTL6A | 1.004980198 (1.000112866~1.009871218) | 0.044906903 |
| DMAP1 | 0.981279467 (0.962003112~1.000942077) | 0.061910025 |
| HDAC2 | 1.013671458 (0.99910518~1.028450102) | 0.065953405 |
| KAT2A | 0.994832077 (0.98934524~1.000349344) | 0.06633056 |
| MAP3K7 | 1.02052085 (0.997845391~1.043711596) | 0.076423733 |
| RUVBL1 | 1.008247836 (0.997518812~1.019092258) | 0.132366149 |
| MORF4L2 | 1.001041217 (0.999652435~1.002431929) | 0.141779543 |
| RUVBL2 | 1.00383985 (0.998340223~1.009369774) | 0.17152648 |
| FOXO1 | 1.008408858 (0.995961805~1.021011467) | 0.186360791 |
| FOXO3 | 1.010179451 (0.994756617~1.025841403) | 0.196968771 |
| BRCA1 | 1.014261668 (0.98693073~1.042349476) | 0.309603343 |
| RBBP4 | 1.003450289 (0.9967856~1.01015954) | 0.311042629 |
| TP53 | 0.996792805 (0.990180882~1.003448878) | 0.344134745 |
| MCRS1 | 1.002982188 (0.99507425~1.010952972) | 0.460935385 |
| MORF4L1 | 1.001793269 (0.996975613~1.006634206) | 0.466337493 |
| ING3 | 0.981743869 (0.928965104~1.037521238) | 0.513433182 |
| EP400 | 0.990458008 (0.962376356~1.019359069) | 0.513526451 |
| HDAC1 | 1.001360884 (0.997208442~1.005530617) | 0.521234077 |
| CHD4 | 1.001187275 (0.997447569~1.004941003) | 0.534302591 |
| POLE3 | 0.998648027 (0.993737013~1.003583312) | 0.590663727 |
| KAT5 | 1.00512915 (0.985931063~1.024701063) | 0.603095519 |
| MLLT3 | 0.993550069 (0.968738353~1.018997273) | 0.616027853 |
| MRGBP | 1.002242152 (0.990098849~1.01453439) | 0.718775377 |
| KAT6B | 1.008021763 (0.964515131~1.05349086) | 0.722636931 |
| KANSL1 | 0.997454956 (0.976011133~1.019369919) | 0.818236115 |
| WDR5 | 0.998931505 (0.987178125~1.010824821) | 0.859481197 |
| KAT2B | 1.001331243 (0.980103019~1.023019252) | 0.903148778 |
| TRRAP | 0.999905835 (0.987358088~1.012613043) | 0.988338958 |

*HR, hazard ratio.*
