# Supplementary material for: Construction and experimental validation of an acetylation-related gene signature to evaluate the recurrence and immunotherapeutic response in early-stage lung adenocarcinoma
Source: BMC Med Genomics. 2022 Dec 11;15:254. doi: 10.1186/s12920-022-01413-7 (PMC9741798; doi:10.1186/s12920-022-01413-7)
Supplement: Supplementary file 9 — Additional file 9. Table S7: The baseline clinicopathological information of the training and external validation cohorts. [file 12920_2022_1413_MOESM9_ESM.docx]

**Additional file 9: Table S7** The baseline clinicopathological information of the training and external validation cohorts.

| Characteristics | | Cohorts | | | | |
| --- | --- | --- | --- | --- | --- | --- |
|  |  | TCGA (n = 334) | GSE31210 (n = 226) | GSE30219 (n = 81) | GSE50081 (n = 121) | GSE37745 (n = 43) |
| Age | |  |  |  |  |  |
|  | <=65 | 156 (46.71%) | 176 (77.88%) | 59 (72.84%) | 40 (33.06%) | 20 (46.51%) |
|  | >65 | 169 (50.60%) | 50 (22.12%) | 22 (27.16%) | 81 (66.94%) | 23 (53.49%) |
|  | Unknown | 9 (2.69%) | 0 (0.00%) | 0 (0.00%) | 0 (0.00%) | 0 (0.00%) |
| Gender | |  |  |  |  |  |
|  | Male | 153 (45.81%) | 105 (46.46%) | 64 (79.01%) | 62 (51.24%) | 18 (41.86%) |
|  | Female | 181 (54.19%) | 121 (53.54%) | 17 (20.99%) | 59 (48.76%) | 25 (58.14%) |
| Smoking | |  |  |  |  |  |
|  | No | 50 (14.97%) | 115 (50.88%) | - | 22 (18.18%) | - |
|  | Yes | 275 (82.34%) | 111 (49.12%) | - | 88 (72.73%) | - |
|  | Unknown | 9 (2.69%) | 0 (0.00%) | - | 11 (9.09%) | - |
| T stage | |  |  |  |  |  |
|  | T1 | 128 (38.32%) | - | 68 (83.95%) | 41 (33.88%) | - |
|  | T2 | 181 (54.19%) | - | 12 (14.81%) | 78 (64.46%) | - |
|  | T3 | 25 (7.49%) | - | 1 (1.23%) | 2 (1.65%) | - |
| N stage | |  |  |  |  |  |
|  | N0 | 264 (79.04%) | - | 79 (97.53%) | 90 (74.38%) | - |
|  | N1 | 65 (19.46%) | - | 2 (2.47%) | 31 (25.62%) | - |
|  | Unknown | 5 (1.50%) | - | 0 (0.00%) | 0 (0.00%) | - |
| TNM Stage | |  |  |  |  |  |
|  | Stage I | 232 (69.46%) | 168 (74.34%) |  | 88 (72.73%) | 33 (76.74%) |
|  | Stage II | 102 (30.54%) | 58 (25.66%) |  | 33 (27.27%) | 10 (23.26%) |
| Recurrence status | |  |  |  |  |  |
|  | Recurrence | 127 (38.02%) | 64 (28.32%) | 26 (32.10%) | 37 (30.58%) | 21 (48.84%) |
|  | Non-recurrence | 207 (61.98%) | 162 (71.68%) | 55 (67.90%) | 84 (69.42%) | 22 (51.16%) |

*The data is presented as n (%).*
